# Supplementary material for: Conformational changes in the human Cx43/GJA1 gap junction channel visualized using cryo-EM
Source: Nat Commun. 2023 Feb 18;14:931. doi: 10.1038/s41467-023-36593-y (PMC9938869; doi:10.1038/s41467-023-36593-y)

Conformational changes in the human Cx43/GJA1 gap junction channel visualized using cryo-EM

Hyuk-Joon Lee[1,†], Hyung Jin Cha[1,†], Hyeongseop Jeong[1,2,†], Seu-Na Lee[1], Chang-Won Lee[1], Minsoo Kim[3], Jejoong Yoo[3], Jae-Sung Woo[1,*]

[1]Department of Life Sciences, Korea University, Seoul 02841, Korea, [2]Center for Research Equipment, Korea Basic Science Institute, Chungcheongbuk-do 28119, Korea, [3]Department of Physics, Sungkyunkwan University, Suwon 16419, Korea

[*]To whom correspondence should be addressed.

Tel: +82-2-3290-3406, Fax: +82-2-927-9028, jaesungwoo@korea.ac.kr (J.-S.W.).

[†]These authors contribute equally to this work.

Key words: cryo-EM, gap junction intercellular channel, conformational change, N-terminal helix, pore size, ion selectivity, π-helix

Running title: Cryo-EM structures of Cx43.

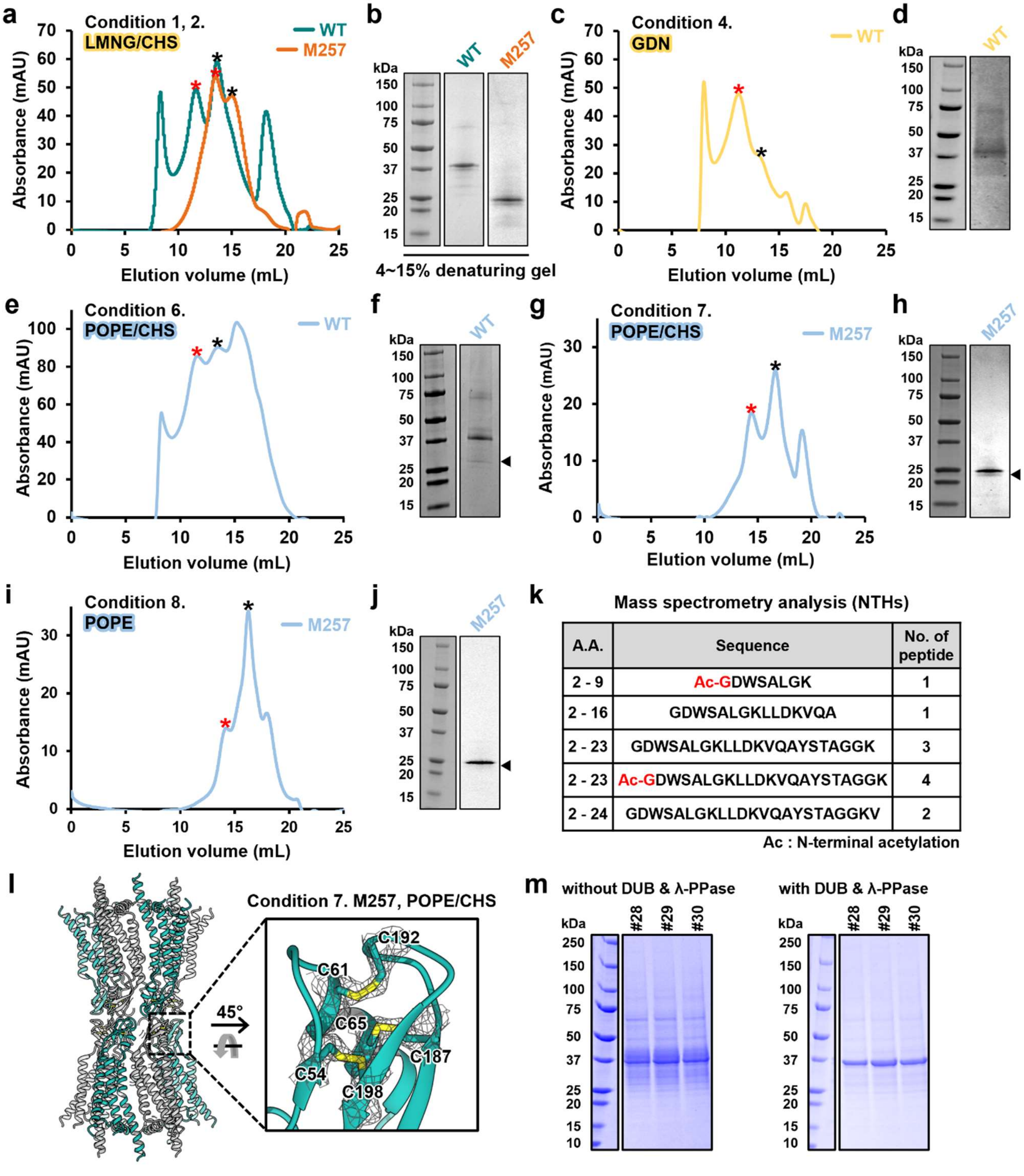

Supplementary Figure 1. Purification of Cx43 GJIChs.

a Gel filtration profiles of Cx43-WT (green) and Cx43-M257 (orange) in LMNG/CHS. Asterisks in red and black indicate peaks presumed to be GJIChs and hemichannels, respectively.

b SDS-PAGE gel of purified Cx43-WT (left) and Cx43-M257 (right) in LMNG/CHS.

c-f Purification of Cx43-WT in GDN and in lipid-nanodiscs at pH 8.0. Gel filtration profiles of Cx43-WT in GDN (**c**) and Cx43-WT in nanodiscs (POPE/CHS) (**e**). Asterisks colored in red and black indicate gel filtration peaks presumed to be GJIChs and hemichannels, respectively. SDS-PAGE gels of purified Cx43-WT in GDN (**d**) and Cx43-WT in POPE/CHS-nanodiscs (**f**). MSP1E3D1 is indicated by an arrowhead.

g-j Purification of Cx43-M257 in lipid-nanodiscs at pH 8.0. Gel filtration profiles of Cx43-M257 in POPE/CHS-nanodiscs (**g**) and Cx43-M257 in POPE-nanodiscs (**i**). SDS-PAGE gels of purified Cx43-M257 in POPE/CHS-nanodiscs (**h**) and Cx43-M257 in POPE-nanodiscs (**j**). MSP1E1 is indicated by an arrowhead.

k Mass spectrometry analysis of purified Cx43-WT. The first methionine residue was not detected in any identified N-terminal peptides. The N-terminal G2 was acetylated (Ac-G) in two peptides and highlighted in red color.

l Ribbon representation of the Cx43-M257 GJICh structure viewed from the membrane plane (left). Close-up view of the boxed region (right). Three disulfide bonds at the ECLs are drawn as sticks, labeled, and shown with the corresponding density maps.

m Comparison between the SDS-PAGE gels of purified Cx43-WT samples with and without the treatment with λ-PPase and M48. See Methods for details.

Experiments for purification in Supplementary Fig. 1b,d,f,h,j,m were replicated more than three times with similar results.

Condition 1. WT, pH 8.0, LMNG/CHS

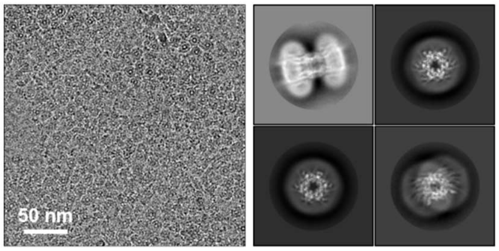

Condition 2. M257, pH 8.0, LMNG/CHS

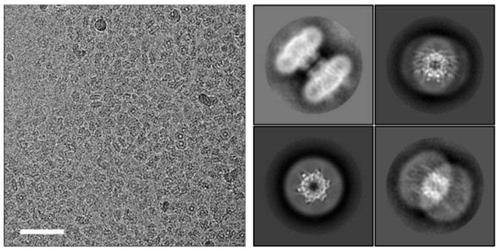

Condition 3. WT, pH 6.9, LMNG/CHS

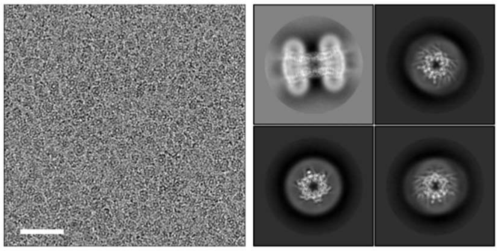

Condition 4. WT, pH 8.0, GDN

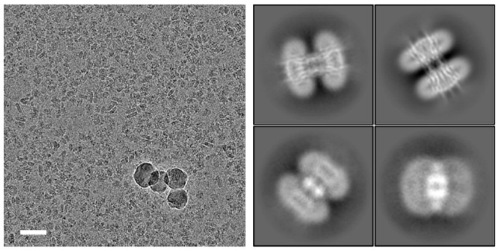

Condition 5. WT, pH 8.0, Soybean lipids

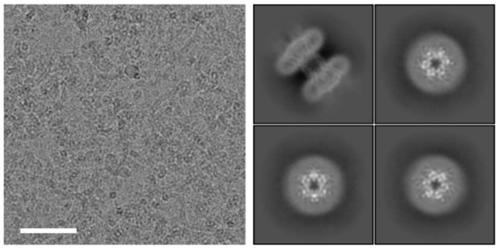

Condition 6. WT, pH 8.0, POPE/CHS

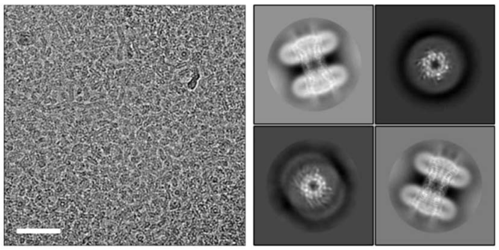

Condition 7. M257, pH 8.0, POPE/CHS

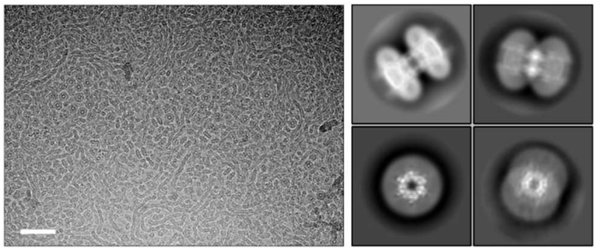

Condition 8. M257, pH 8.0, POPE

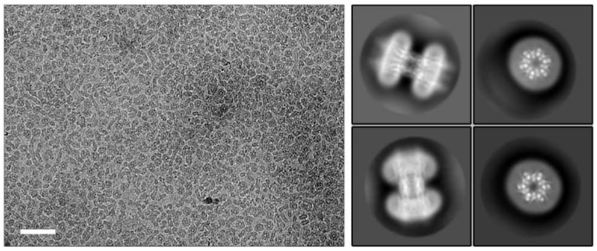

Supplementary Figure 2. Representative micrographs and 2D class averages.

A representative micrograph and four 2D class averages are shown for each of datasets 1 to 8. More than two thousand micrographs in each dataset showed similar results. A 50 nm scale bar is shown in each micrograph. For datasets 1 to 8, particles in 2D class averages were extracted into 400, 400, 400, 256, 512, 420, 320, and 320 pixel boxes, respectively.

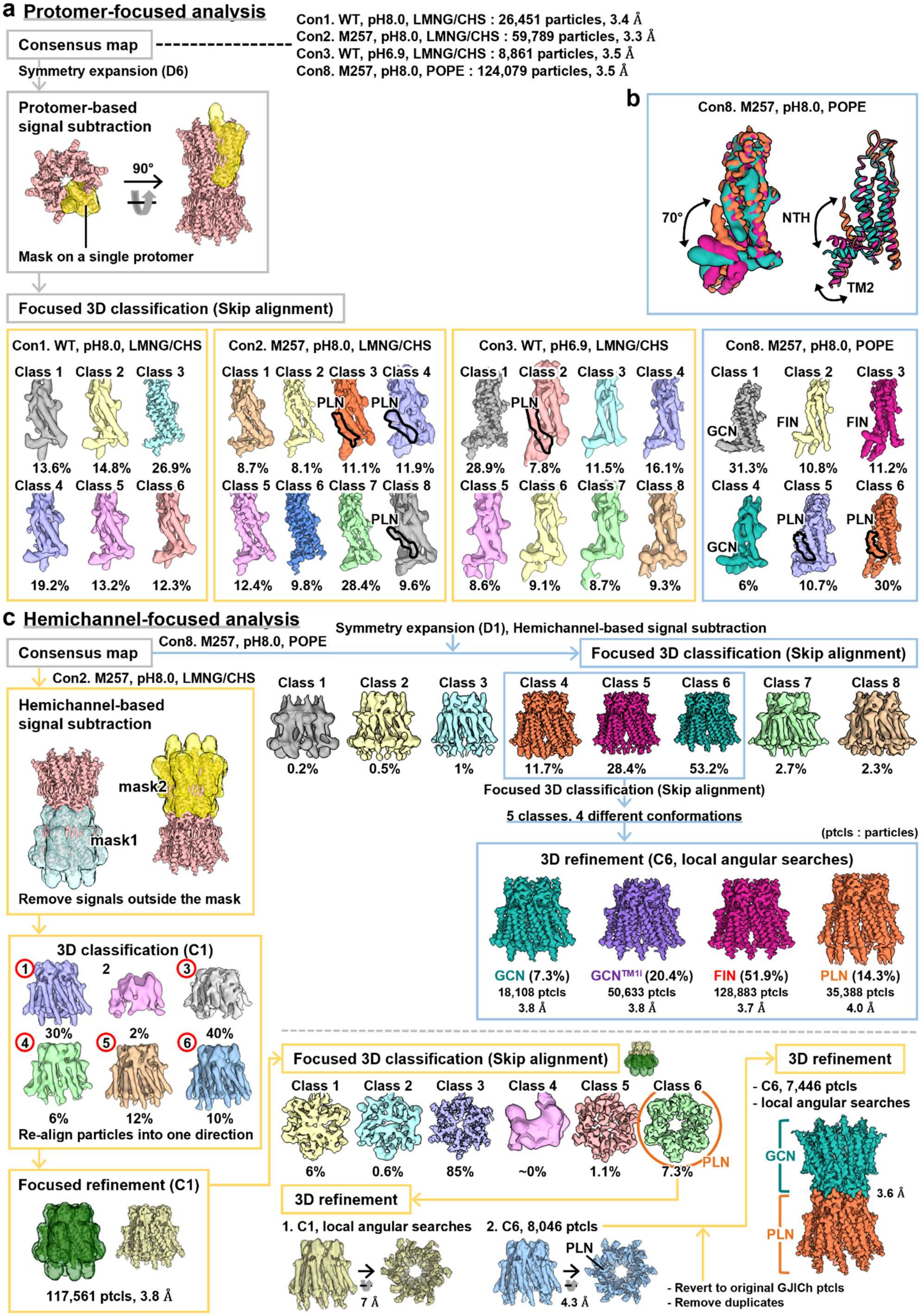
a Protomer-focused analysis
Consensus map
Con1. WT, pH8.0, LMNG/CHS : 26,451 particles, 3.4 Å
Con2. M257, pH8.0, LMNG/CHS : 59,789 particles, 3.3 Å
Con3. WT, pH6.9, LMNG/CHS : 8,861 particles, 3.5 Å
Con8. M257, pH8.0, POPE : 124,079 particles, 3.5 Å
Symmetry expansion (D6)
Protomer-based signal subtraction
90°
Mask on a single protomer
b
Con8. M257, pH8.0, POPE
70°
NTH
TM2
Focused 3D classification (Skip alignment)
Con1. WT, pH8.0, LMNG/CHS
Class 1 Class 2 Class 3
13.6% 14.8% 26.9%
Class 4 Class 5 Class 6
19.2% 13.2% 12.3%
Con2. M257, pH8.0, LMNG/CHS
Class 1 Class 2 Class 3 Class 4
PLN PLN
8.7% 8.1% 11.1% 11.9%
Class 5 Class 6 Class 7 Class 8
PLN
12.4% 9.8% 28.4% 9.6%
Con3. WT, pH6.9, LMNG/CHS
Class 1 Class 2 Class 3 Class 4
PLN
28.9% 7.8% 11.5% 16.1%
Class 5 Class 6 Class 7 Class 8
8.6% 9.1% 8.7% 9.3%
Con8. M257, pH8.0, POPE
Class 1 Class 2 Class 3
GCN FIN FIN
31.3% 10.8% 11.2%
Class 4 Class 5 Class 6
GCN PLN PLN
6% 10.7% 30%
c Hemichannel-focused analysis
Symmetry expansion (D1), Hemichannel-based signal subtraction
Consensus map
Con8. M257, pH8.0, POPE
Focused 3D classification (Skip alignment)
Class 1 Class 2 Class 3 Class 4 Class 5 Class 6 Class 7 Class 8
0.2% 0.5% 1% 11.7% 28.4% 53.2% 2.7% 2.3%
Focused 3D classification (Skip alignment)
5 classes. 4 different conformations
(ptcls : particles)
3D refinement (C6, local angular searches)
GCN (7.3%) 18,108 ptcls 3.8 Å
GCN^{TM1I} (20.4%) 50,633 ptcls 3.8 Å
FIN (51.9%) 128,883 ptcls 3.7 Å
PLN (14.3%) 35,388 ptcls 4.0 Å
Con2. M257, pH8.0, LMNG/CHS
Hemichannel-based signal subtraction
mask2
mask1
Remove signals outside the mask
3D classification (C1)
1 2 3
30% 2% 40%
4 5 6
6% 12% 10%
Re-align particles into one direction
Focused refinement (C1)
117,561 ptcls, 3.8 Å
Focused 3D classification (Skip alignment)
Class 1 Class 2 Class 3 Class 4 Class 5 Class 6
6% 0.6% 85% ~0% 1.1% 7.3%
PLN
3D refinement
1. C1, local angular searches
7 Å
2. C6, 8,046 ptcls
PLN
4.3 Å
3D refinement
- C6, 7,446 ptcls
- local angular searches
GCN
PLN
3.6 Å
- Revert to original GJICh ptcls
- Remove duplicates

Supplementary Figure 3. Workflow of cryo-EM image processing using protomer-focused and hemichannel-focused analyses.

a Workflow of cryo-EM image processing using focused classification on a protomer of Cx43-WT and Cx43-M257 in four conditions. The densities of NTH in the PLN conformation are indicated by a black bold line. See Methods for details.

b Structural alignment between three different protomer conformations of Cx43-M257 in nanodiscs (POPE) at pH 8.0. Movements of NTH and TM2 are indicated by two-way arrows.

c Workflow of cryo-EM image processing of Cx43-M257 GJICh including hemichannel-focused classification and localized reconstruction. Five red circles and an orange circle indicate the selected particle classes for the next process. Transparent blue, yellow and green masks indicate the regions selected for reconstruction and classification. See Methods for details.

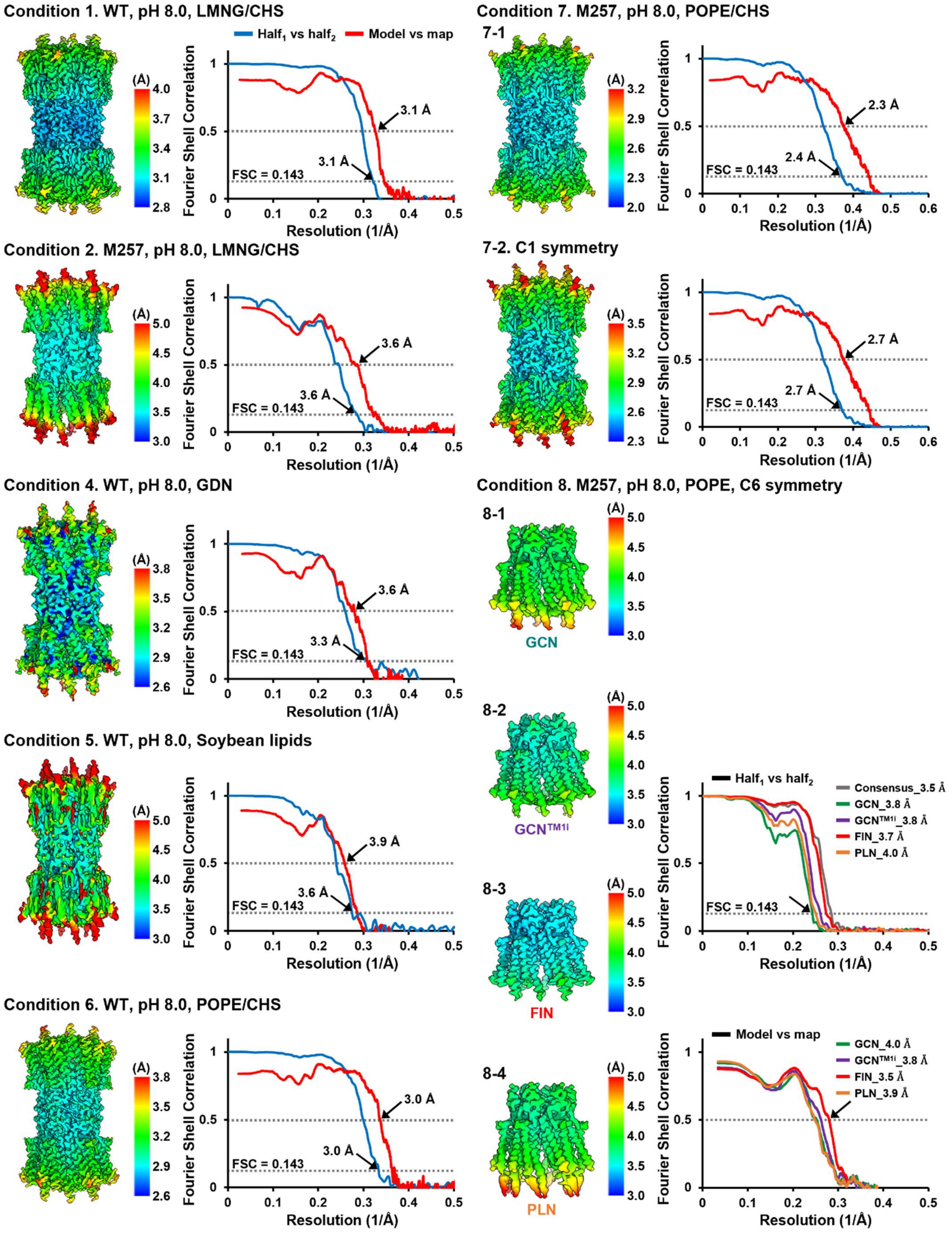
Condition 1. WT, pH 8.0, LMNG/CHS
Half1 vs half2
Model vs map
Fourier Shell Correlation
Resolution (1/Å)
FSC = 0.143
3.1 Å
3.1 Å
Condition 2. M257, pH 8.0, LMNG/CHS
3.6 Å
3.6 Å
Condition 4. WT, pH 8.0, GDN
3.6 Å
3.3 Å
Condition 5. WT, pH 8.0, Soybean lipids
3.9 Å
3.6 Å
Condition 6. WT, pH 8.0, POPE/CHS
3.0 Å
3.0 Å
Condition 7. M257, pH 8.0, POPE/CHS
7-1
2.3 Å
2.4 Å
7-2. C1 symmetry
2.7 Å
2.7 Å
Condition 8. M257, pH 8.0, POPE, C6 symmetry
8-1
GCN
8-2
GCNTM1i
8-3
FIN
8-4
PLN
Half1 vs half2
Consensus_3.5 Å
GCN_3.8 Å
GCNTM1i_3.8 Å
FIN_3.7 Å
PLN_4.0 Å
Model vs map
GCN_4.0 Å
GCNTM1i_3.8 Å
FIN_3.5 Å
PLN_3.9 Å

Supplementary Figure 4. Validation of cryo-EM reconstruction maps.

Validation of cryo-EM maps of Cx43-WT and Cx43-M257 in various conditions. Cryo-EM maps colored according to local resolution estimated by ResMap (left). The resolution range bars indicate local resolution gradients. Fourier Shell Correlation (FSC) curves between the two unfiltered half maps and between the model and the full map (right). For Cx43-M257 in POPE/CHS-nanodiscs (condition 7), the 3D structure with C1 symmetry is also presented. For Cx43-M257 in POPE-nanodiscs (condition 8), hemichannel sub-structures in four conformations are presented. If an atomic model is available, FSC curve between the model and the full map is also depicted. FSC curves between the two unfiltered half maps and between the model and the full map are calculated based on the 0.143 and 0.5 FSC criterion, respectively.

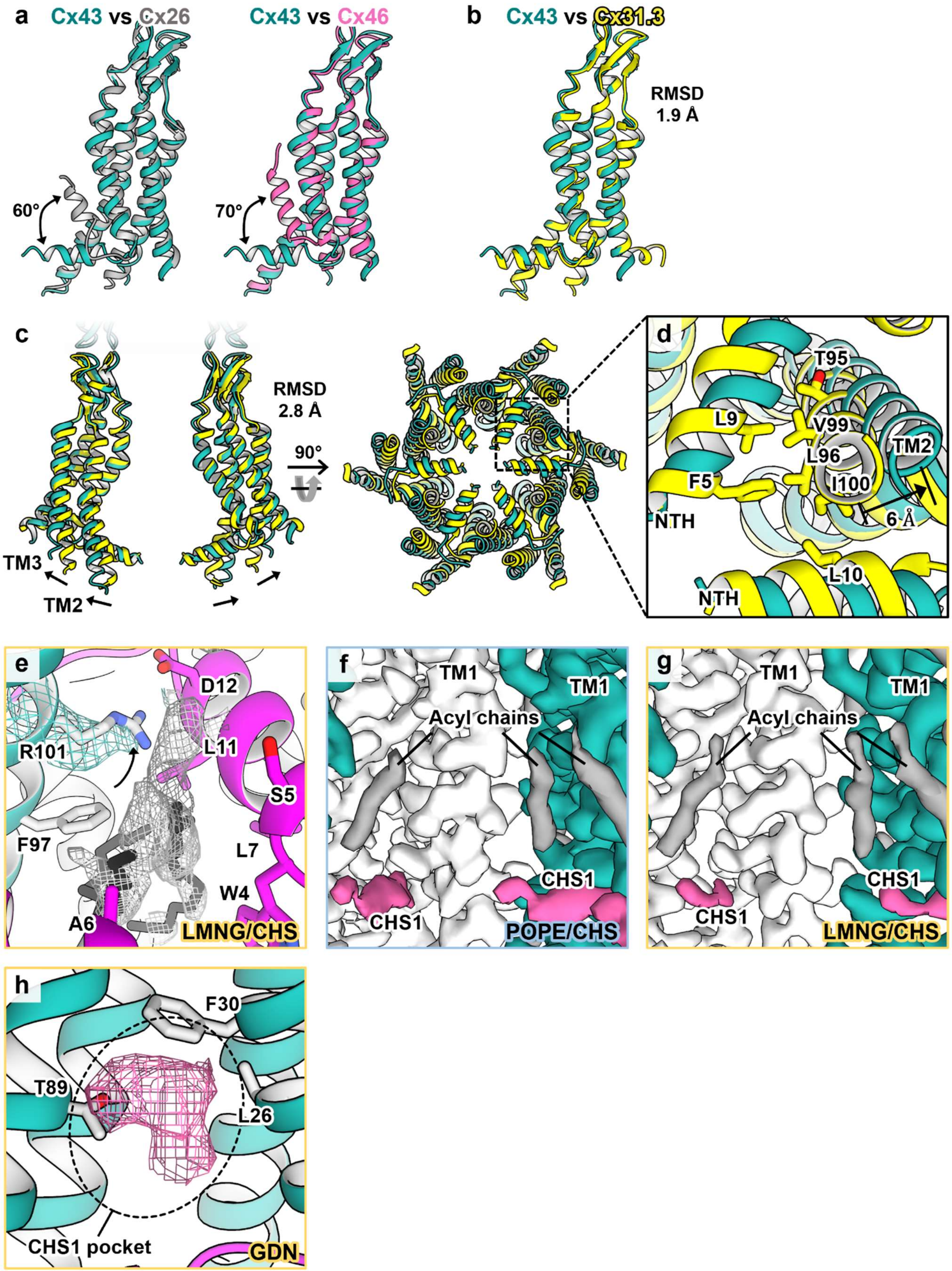
a
Cx43 vs Cx26
Cx43 vs Cx46
60°
70°
b
Cx43 vs Cx31.3
RMSD
1.9 Å
c
RMSD
2.8 Å
90°
TM3
TM2
d
T95
L9
V99
L96
TM2
F5
I100
NTH
6 Å
L10
NTH
e
D12
R101
L11
S5
F97
L7
W4
A6
LMNG/CHS
f
TM1
TM1
Acyl chains
CHS1
CHS1
POPE/CHS
g
TM1
TM1
Acyl chains
CHS1
CHS1
LMNG/CHS
h
F30
T89
L26
CHS1 pocket
GDN

Supplementary Figure 5. Structural comparison of Cx43 with other connexins and comparison between lipid-binding modes in different conditions.

a Superposition of the Cx43 protomer (green) in the GCN state with the Cx26 protomer (gray; PDB accession code 2ZW3; left) and Cx46 (pink; PDB accession code 7JKC; right). Two-way arrows indicate conformational differences of NTHs between two superposed connexins.

b Superposition of the Cx43 protomer in the GCN state with the Cx31.3 protomer (yellow; PDB accession code 6L3T).

c Superposition of a hemichannel (green) in the full GCN structure of Cx43 GJICh with the Cx31.3 hemichannel (yellow). For superposition of the two models, the Cx31.3 hemichannel model was fit in the Cx43 GJICh map. The superposed structures are viewed from the membrane (left) and the cytoplasm (right). In the view from the membrane, only two protomers facing each other are shown for clarity. One-way arrows indicate conformational differences in the cytoplasmic regions of TM2 and TM3.

d Close-up view of the space between two neighboring NTHs and TM2. Residues involved in the intramolecular NTH-TM2 interaction in the GCN state of Cx31.3 hemichannel are drawn as sticks and labeled. An arrow indicates substantial difference in the TM2 position between Cx43 and Cx31.3.

e Map density of LMNG in Cx43-WT GJICh in LMNG/CHS (condition 1).

f,g Similar binding modes of acyl chains (gray) and CHS1 (pink) in Cx43 GJIChs in POPE/CHS-nanodiscs (condition 7) (**f**) and in LMNG/CHS detergents (condition 1) (**g**).

h Close-up view of the CHS1-binding pocket of Cx43-WT GJICh in GDN detergents (condition 4).

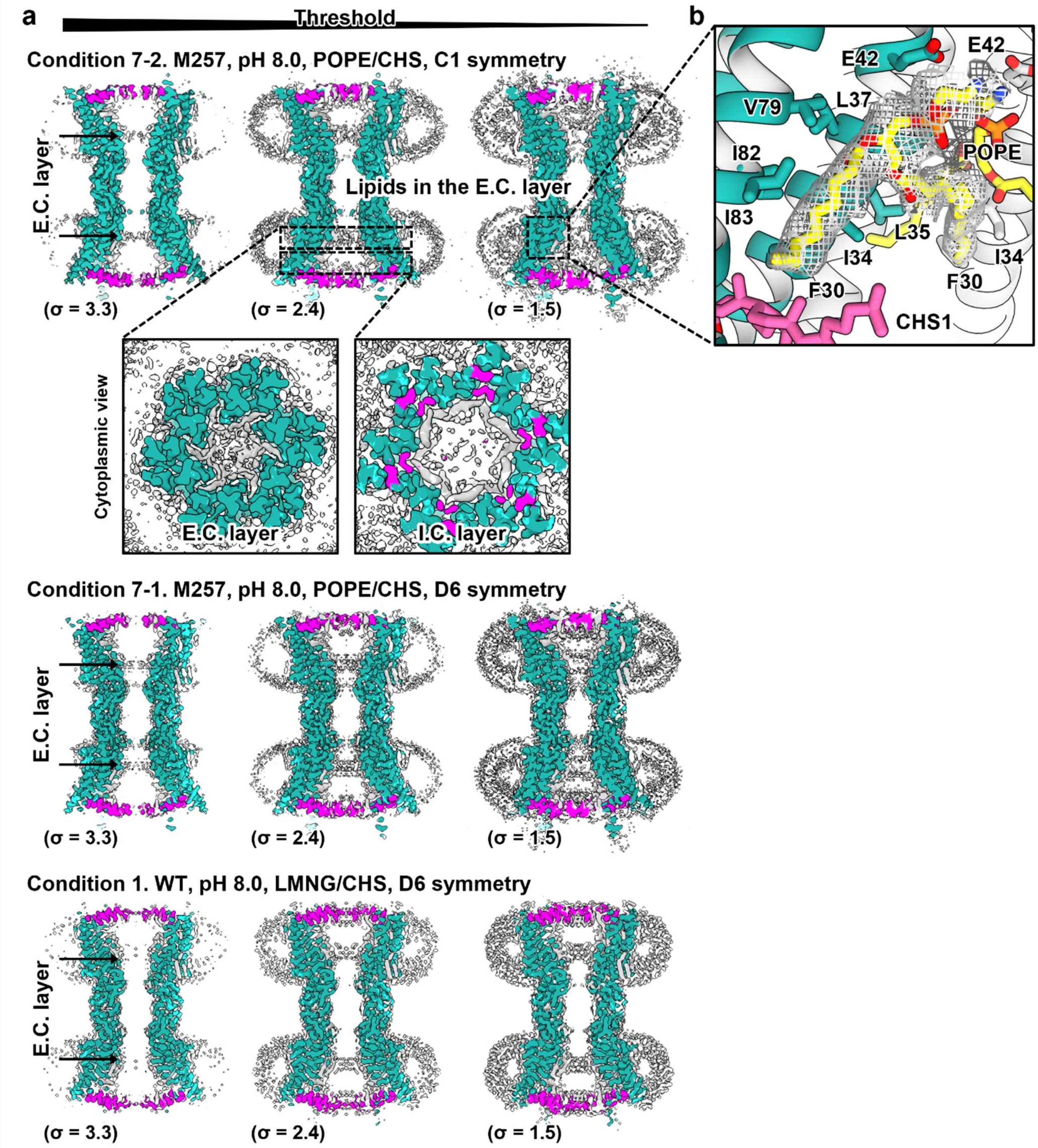

Supplementary Figure 6. Pore-occlusion by lipids in the structures in the full GCN conformation.

a Sliced views of the 3D map of Cx43 GJICh under conditions 1 and 7, with three different contour levels. Map densities for protein and NTH are colored in green and magenta, respectively. Extracellular layers (E.C. layers) of pore-occluding lipids are indicated by arrows. For the map processed with C1 symmetry imposition (condition 7-2), close-up views of the

intracellular (I.C.) and E.C. layers viewed from the cytoplasmic side are shown at the same contour level.

b Close-up view of the indicated E.C. layer region. A bound POPE and the corresponding density are shown as yellow sticks and gray mesh. Residues in close contact with POPE are drawn as sticks and labeled.

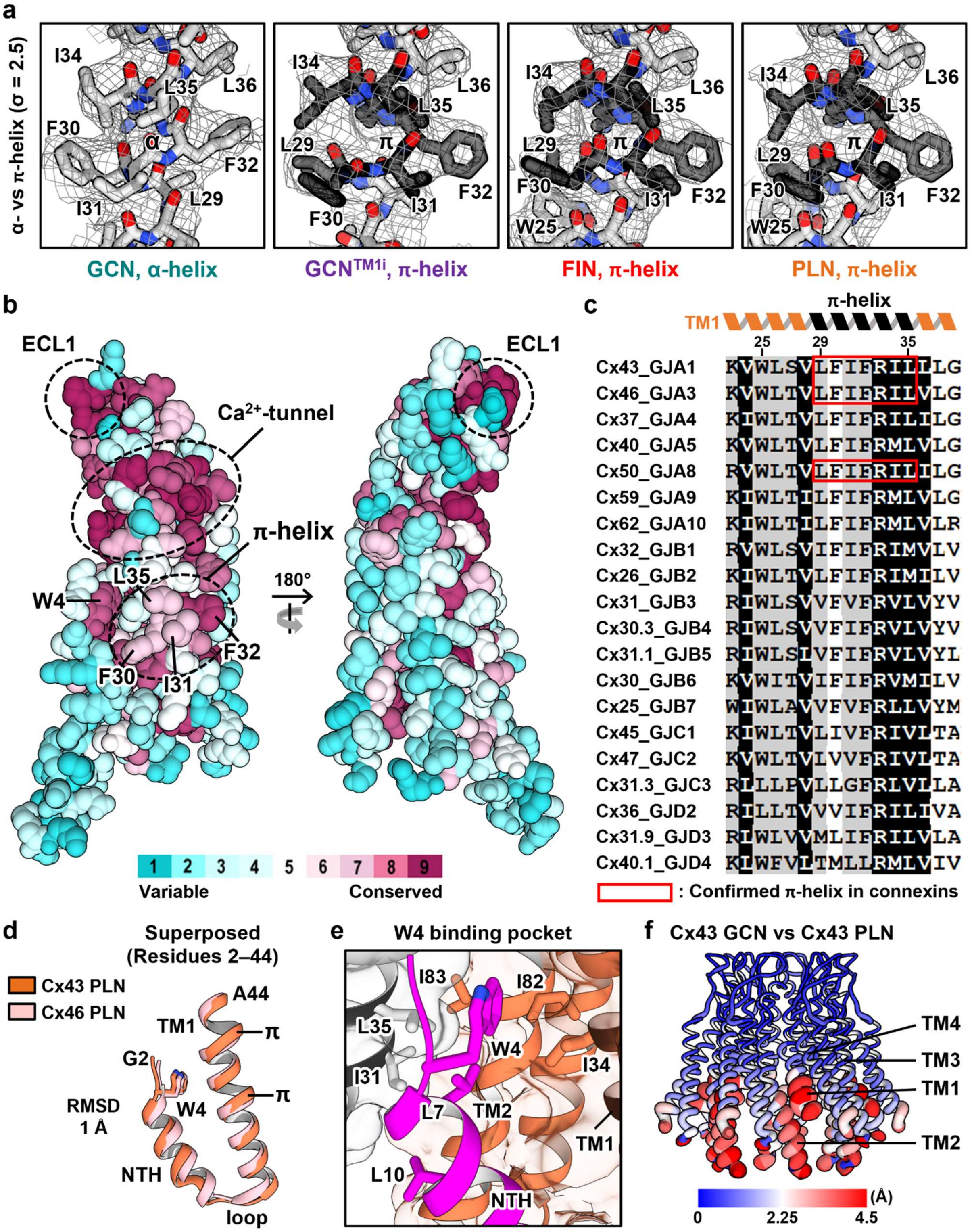

Supplementary Figure 7. Formation and conservation of π-helix in the middle of TM1.

a Structural comparison of the middle part of TM1 between Cx43 GJICh in GCN, GCN^{TM1i}, FIN, and PLN conformation. π-helices are colored in black.

b Consurf analysis of Cx43. On a space filling model of Cx43 in the PLN state, amino acids are colored based on the degree of sequence conservation among 20 human connexin proteins, except the highly diversified member Cx23, from the most conserved (purple) to the least conserved (cyan). Three most conserved regions are indicated by dotted circles and labeled.

c Sequence alignment of TM1s from 20 human connexin proteins. The π-helix-forming sequences confirmed by Cx43, Cx46, and Cx50 structures are indicated by red boxes. 100% conserved positions are shaded in black, and >80% similarly conserved positions are in gray, respectively.

d Structural similarity between Cx43 (left) and Cx46 (PDB accession code 7JKC; middle) in the PLN state. Only the region from NTH to TM1 is shown and compared for clarity. Two π-helices in the extracellular end and the middle of TM1 are indicated by lines and labeled in each of Cx43 and Cx46 structures. Cα root mean square deviation (RMSD) between the two superposed structures (right) is 1 Å.

e Detailed view of 'W4 binding pocket'. 'W4 binding pocket' is formed by two TM1s and a TM2 at the interface between two neighboring protomers. On the ribbon drawings of NTH (magenta), TM1 and TM2 in one protomer (orange), and TM1 in the other protomer (gray), W4-interacting residues are drawn as sticks and labeled.

f Structural alignments of Cx43-WT hemichannel region (GCN conformation) with Cx43-M257 hemichannel region (PLN conformation). Cα deviation of each residue is colored according to the range bar at the bottom. NTHs are not included in the calculation of Cα deviation.

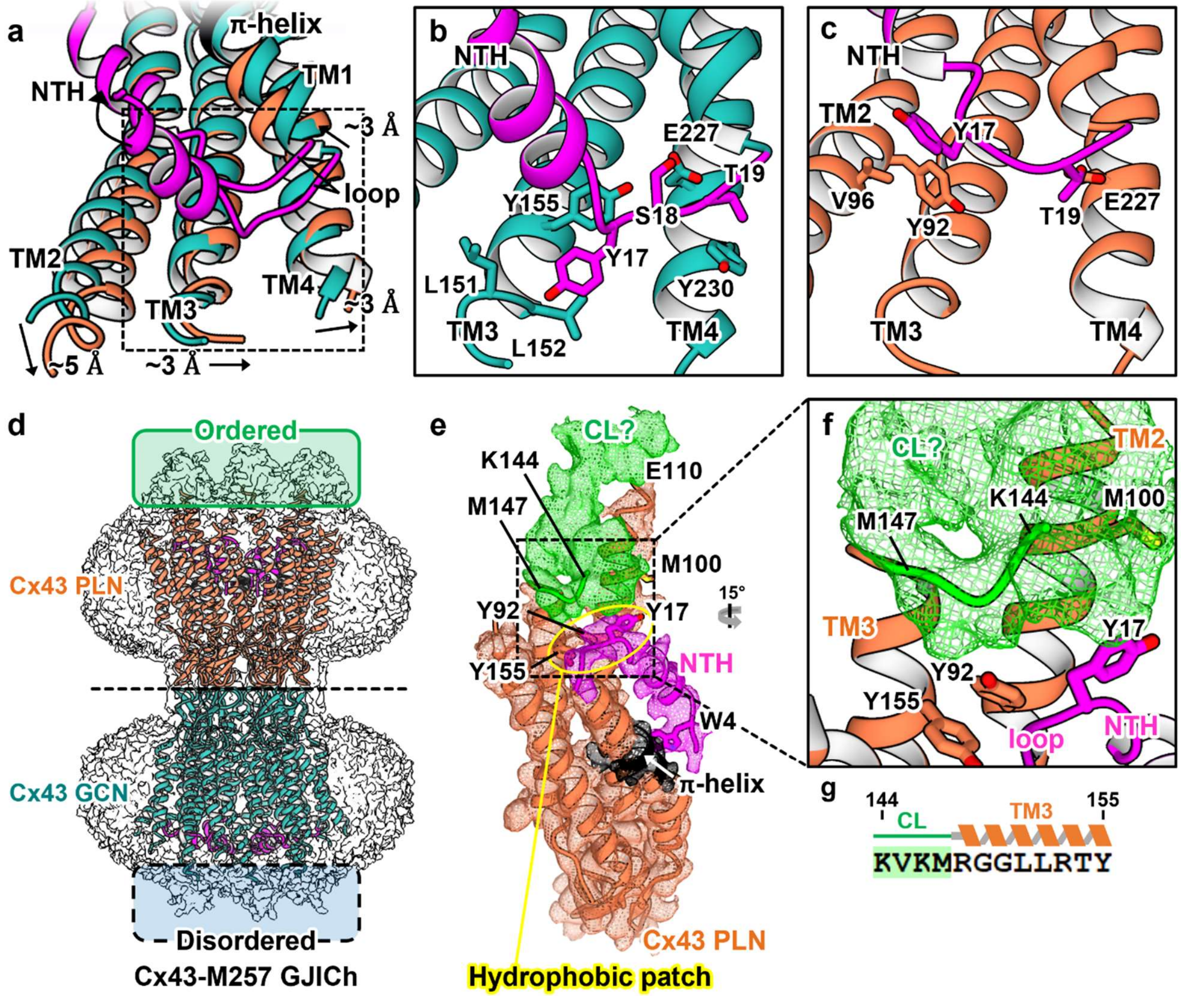

Supplementary Figure 8. Structural comparison of NTH-TM1 loops and TM helices between the GCN and PLN conformations.

a Superposition of GCN (green) and PLN (orange) protomers of Cx43 GJICh shows the conformational change of the NTH-TM1 loop. Arrows indicate conformational changes of NTH (magenta) and TM helices. The moving distances of the cytoplasmic ends of four TM helices are shown next to arrows.

b,c Detailed structural comparison between GCN (**b**) and PLN (**c**) protomers at the boxed region in **a**. Residues participating in the interactions between loop and TMs are drawn as sticks and labeled.

d Map densities observed in the PLN hemichannel region (orange) of heterotypic channel, but not in the GCN hemichannel region (green) due to the structural disorder.

e The ribbon model of Cx43 in PLN conformation is shown with its cryo-EM map density

(mesh). NTH, CL, π-helix and the rest are colored in magenta, green, black and orange, respectively. E110 and M147 at the N- and C-terminus of CL are indicated and labeled. The residues that closely interact with the C-terminal region of CL (Y17 in the NTH-TM1 loop, Y92 in TM2, M100 in TM2, and Y155 in TM3) are drawn as sticks and labeled.

f Close-up view of the region boxed in **e**.

g Sequence of residues 144–155 of Cx43.

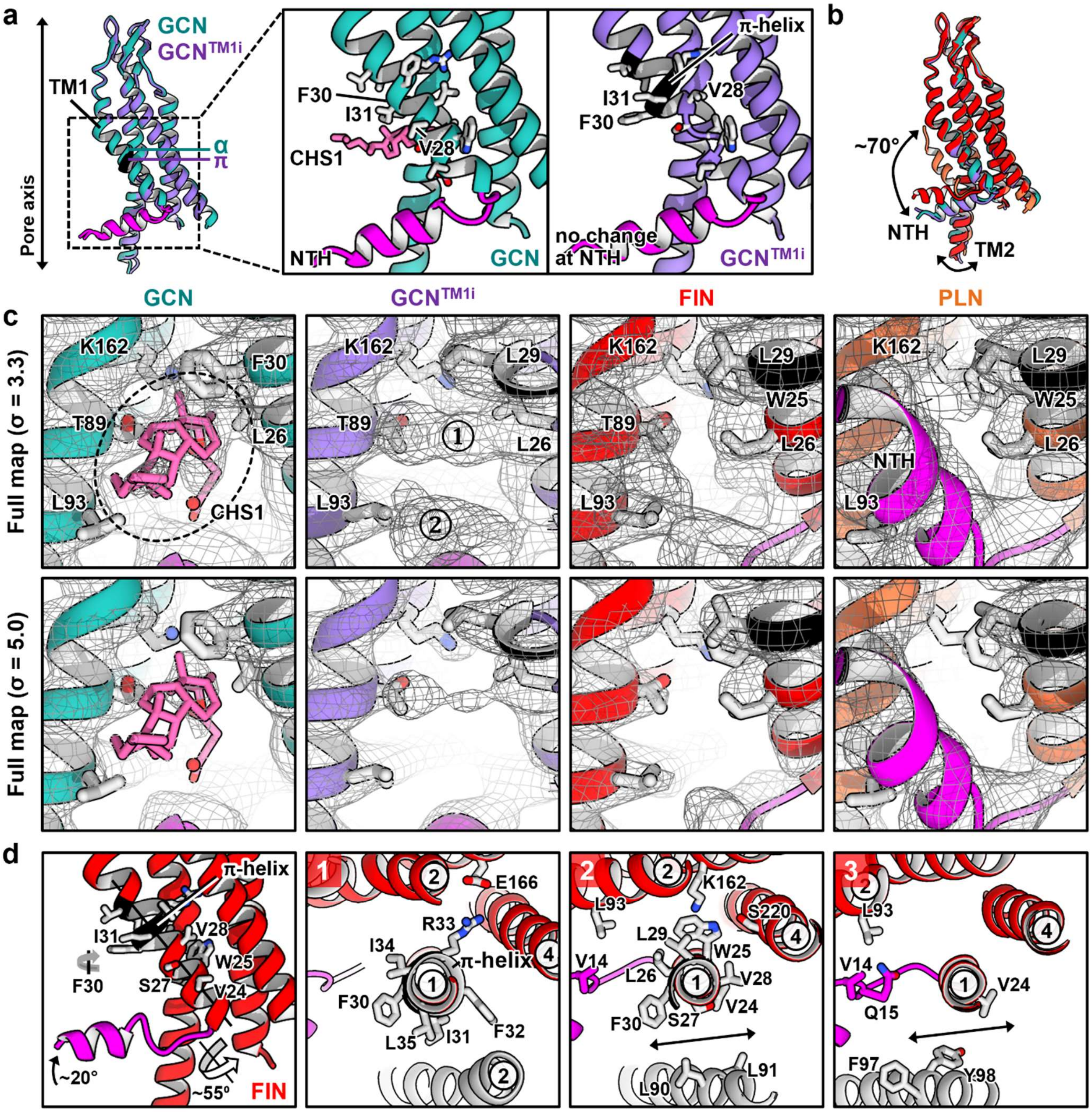

Supplementary Figure 9. Comparison between GCN, GCN^{TM1i}, FIN, and PLN hemichannel sub-structures.

a Superposition of GCN and GCN^{TM1i} protomers in the same orientation as shown in Fig. 5a. In the close-up view of the boxed region, CHS1 and the residues undergoing large conformational changes are drawn as sticks and labeled.

b Superposition of GCN, GCN^{TM1i}, FIN, and PLN protomers. The movements of NTH and TM2 are indicated by two-way arrows.

c Comparison of CHS1-binding sites in four different hemichannel sub-structures. Map densities (gray) are shown at two different contour levels (σ = 3.3 and 5.0). CHS1 and the residues in close contact with CHS1 are drawn as sticks and labeled.

d The structure of FIN protomer is represented similarly as in **a**. FIN protomer was cross-sectioned at three levels along the helical axis of TM1 as shown in Fig. 5c. The cross-sections are individually viewed from the extracellular side. TM2 in the neighboring protomer is colored gray. Circled numbers indicates the TM helix numbers.

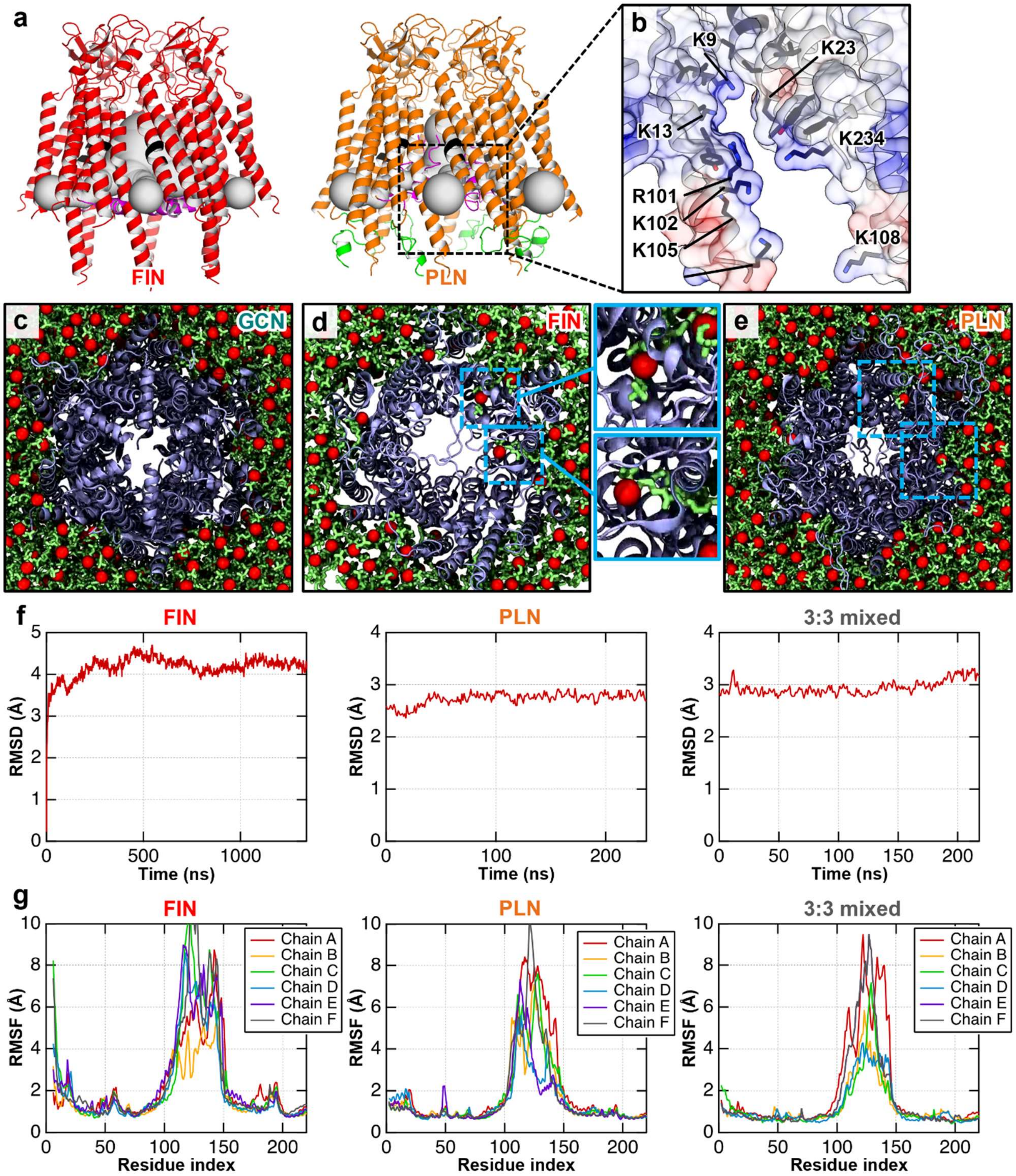

Supplementary Figure 10. Membrane openings in the FIN and PLN hemichannel sub-structures.

a Ribbon representation of FIN hemichannel (left; red) and PLN hemichannel (right; orange) sub-structures of Cx43-M257. Tunnels through the membrane opening are computed by CAVER v3.03 and shown as gray spheres.

b Electrostatic surface potential of the membrane opening. On a transparent surface of the intermolecular interface, the electrostatic potential is colored from red (−10 kT/*e*) to blue (+10 kT/*e*). Positively charged side chains are drawn as sticks and labeled. Hydrophobic residues are also drawn as sticks but not labeled.

c–e MD simulations show partial penetration of phospholipids through membrane openings in FIN (**d**) and PLN (**e**) hemichannels, but not in GCN hemichannel (**c**). Close-up views of membrane openings with penetrating lipids in FIN hemichannel are shown in boxes.

f The graphs represent Cα RMSDs of FIN (left), PLN (middle) and 3:3 mixed (right) hemichannel sub-structures as functions of time from the starting model positions.

g Residue-averaged root mean square fluctuation (RMSF) for FIN (left), PLN (middle) and 3:3 mixed (right) hemichannel sub-structures. The average RMSF value was calculated for each chain in the indicated hemichannel sub-structure.

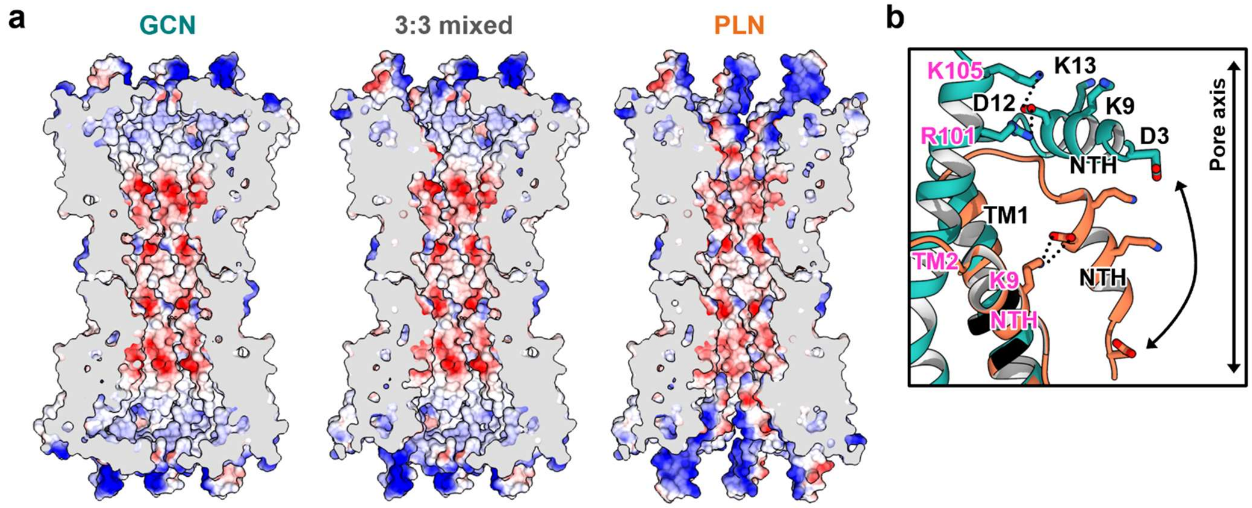

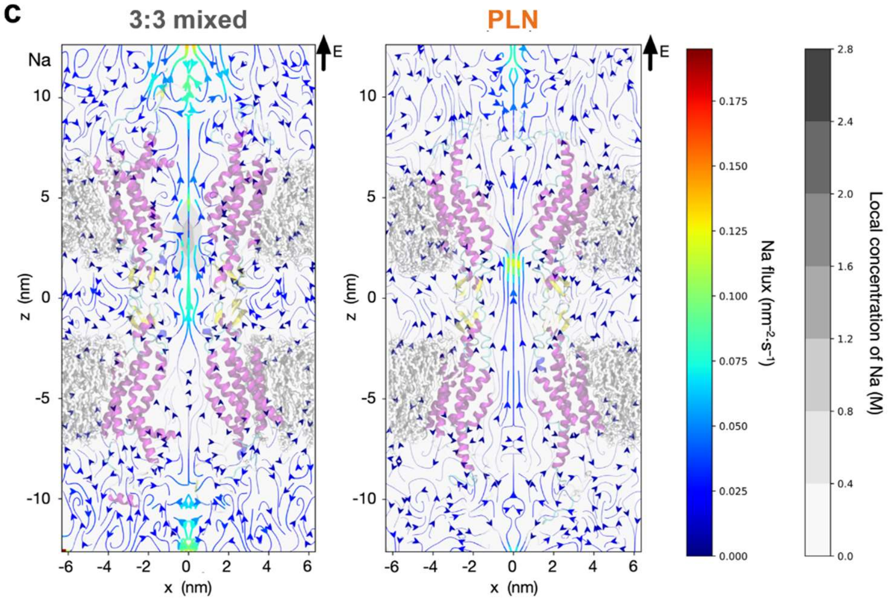

	Na⁺ current (pA)	
Trajectories	3:3 mixed	PLN
0–50 ns	42.4	17.7
50–100 ns	35.5	10.2
100–150 ns	6.1	5.3
150–200 ns	7.2	21.3

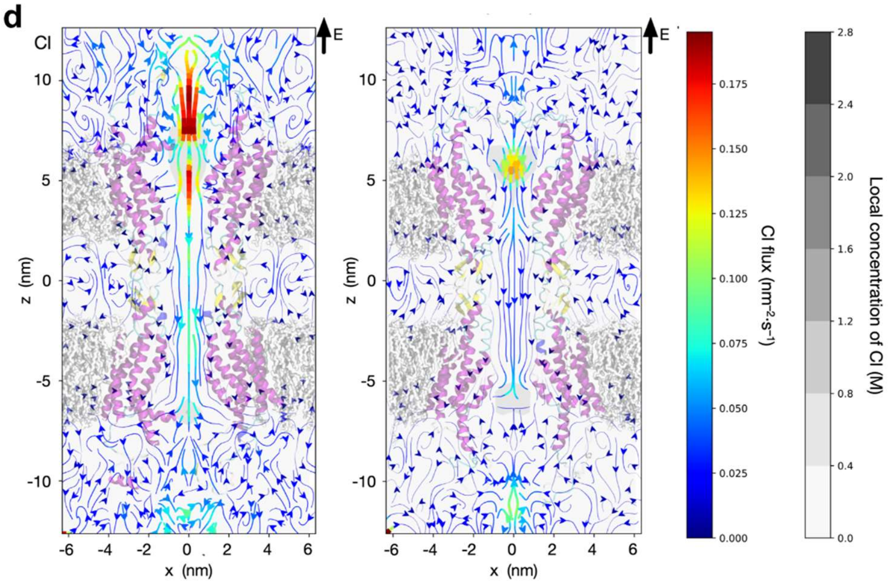

	Cl⁻ current (pA)	
Trajectories	3:3 mixed	PLN
0–50 ns	46.5	-14
50–100 ns	46	5
100–150 ns	37.1	17
150–200 ns	-8.6	-3.2

Supplementary Figure 11. MD simulations of ion transfer through the 3:3 mixed and full PLN GJIChs.

a Electrostatic surface potentials of full GCN, 3:3 mixed, and full PLN models viewed from the membrane.

b GCN and PLN protomers were superposed. Only NTHs, TM1s and TM2s are represented for clarity. D3, K9, D12, and K13 in NTHs are shown as sticks and labeled. Interacting residues in the neighboring protomers are also shown as sticks and labeled in magenta.

c,d Local concentration maps and ionic fluxes of Na (**c**) and Cl (**d**) for 3:3 mixed and full PLN GJIChs. No lipid is included inside the pore pathways. MD simulations are conducted at a 200-mV transmembrane potential. Local concentrations of ions are represented as gray heat maps according to the gray scale indication bar on the right. Ionic fluxes are represented by arrows colored according to the flux intensity scale bar on the right. The ionic currents measured for each range of trajectories are shown in the right panel.

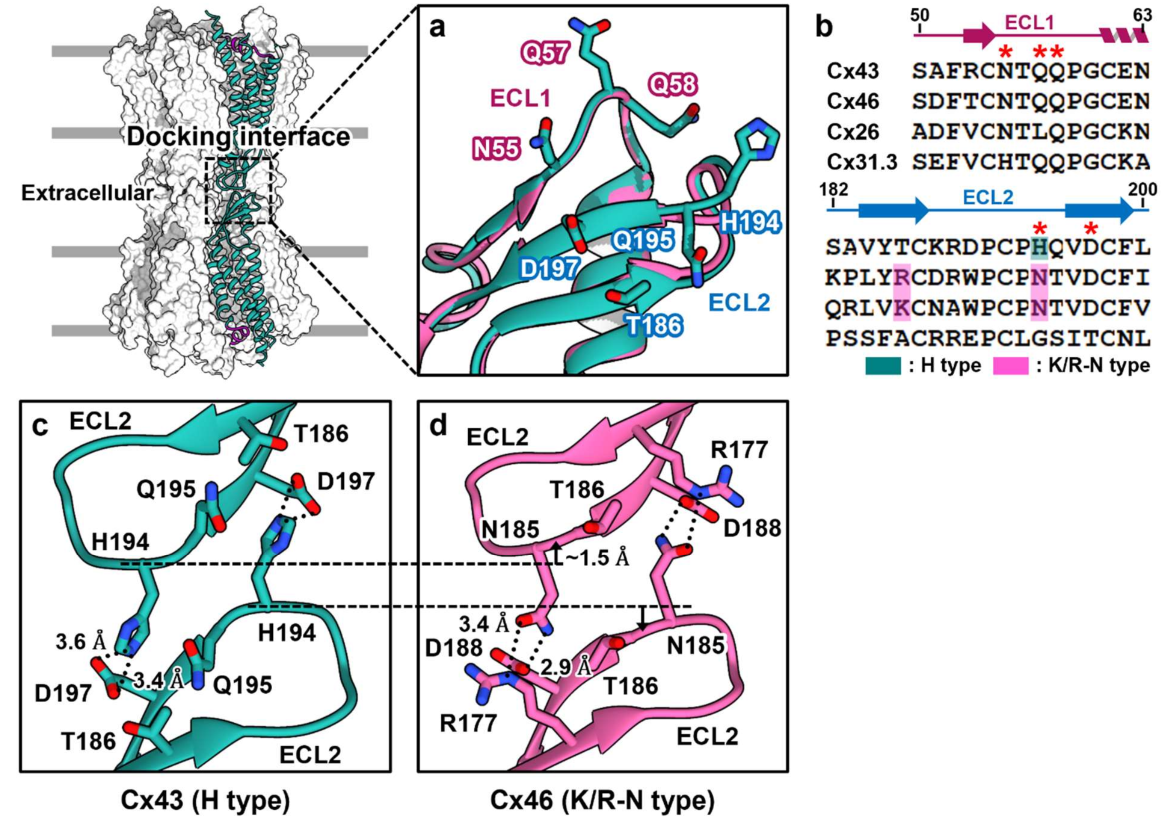

Supplementary Figure 12. Structural comparison of the interconnexon docking interfaces between Cx43 and Cx46 GJIChs.

a Superposition of ECLs of Cx43 (green) and Cx46 (pink) represented as ribbon diagrams. Residues involved in interconnexon interactions of Cx43 GJICh are shown as sticks and labeled.

b Sequence alignment of the ECL1 and ECL2 regions in Cx43, Cx46, Cx26 and Cx31.3. The residues involved in the interconnexon interactions in H type and K/R-N type are shaded by green and pink, respectively. The residues involved in the interconnexon interactions of Cx43 GJICh are indicated by red asterisks.

c,d Comparison of ECL2 docking interfaces of Cx43 (H type; green) (**c**) and Cx46 (K/R-N type; pink) (**d**). Residues in the docking interface are drawn as sticks and labeled. Hydrophilic interactions are represented as dotted line. Arrows indicate the difference between Cα positions of H194 in Cx43 and the corresponding residue N185 in Cx46.

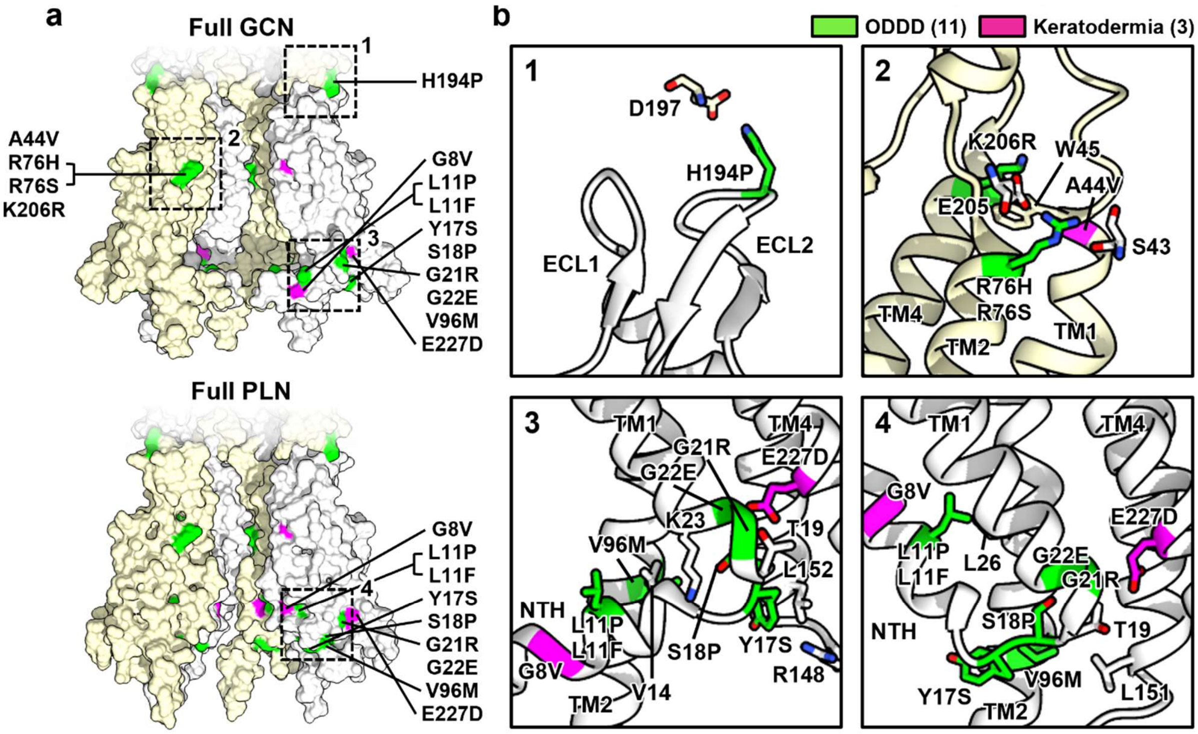

c

Mutations	Putative effect on GCN	Putative effect on PLN
H194P	Negative effect on interconnexon docking interactions	
A44V	Increased hydrophobic interaction with F209	
R76H	Negative effect on intermolecular assembly interactions	
R76S	Negative effect on intermolecular assembly interactions	
K206R	Effect on interaction with W45	
G8V	Effect on lipid-binding	Increased hydrophobic interactions with TM1
L11P	Negative effect on structural integrity	
L11F	Effect on lipid-binding	Increased hydrophobic interactions with TM1
Y17S	Decreased hydrophobic interactions with TM3	Decreased hydrophobic interactions with TM2
S18P	Negative effect on structural integrity	
G21R	Negative effect on structural integrity	
G22E	Negative effect on structural integrity	
V96M	Effect on interactions with NTH	Effect on interactions with NTH-TM1 loop
E227D	Decreased interaction with T19	

Supplementary Figure 13. Location and putative roles of disease-causing mutations in the full GCN and PLN GJIChs.

a Mapping of disease-causing mutations on the surface representation of full GCN (upper) and full PLN (lower) GJIChs. Out of 25 selected mutations in OMIM database

(https://omim.org/allelicVariants/121014), 14 missense mutations were selected and represented. Mutations causing ODDD and keratoderma are colored in green and magenta, respectively. Only four protomers in each hemichannel region are shown for clarity.

b Close-up views of the regions boxed in **a**. The residues corresponding to ODDD and keratoderma mutations are labeled and shown as green and magenta sticks, respectively. Some residues closely interacting with the residues corresponding to the mutations are shown as gray sticks.

c Putative effects of 14 mutations.

Supplementary Table 1. Cryo-EM data collection, refinement and validation statistics

	Cx43-WT					Cx43-M257						
	1	3	4	5	6	2	7-1	7-2	8-1	8-2	8-3	8-4
	GCN, pH 8.0<br><br>In LMNG/CHS<br>EMD-31495<br>PDB 7F92	GCN/PLN, pH 6.9, Consensus<br>In LMNG/CHS<br>EMD-33399	GCN, pH 8.0<br><br>In GDN<br>EMD-33391<br>PDB 7XQ9	GCN, pH 8.0<br><br>In Soy lipids<br>EMD-31496<br>PDB 7F93	GCN, pH 8.0<br><br>In POPE/CHS<br>EMD-33392<br>PDB 7XQB	GCN/PLN, pH 8.0, Heterotypic<br>In LMNG/CHS<br>EMD-31497<br>PDB 7F94	GCN, pH 8.0<br><br>In POPE/CHS<br>EMD-33394<br>PDB 7XQF	GCN, pH 8.0<br><br>In POPE/CHS<br>EMD-33393<br>PDB 7XQD	GCN, pH 8.0<br><br>In POPE<br>EMD-33395<br>PDB 7XQG	GCN^{TM1i}, pH 8.0<br><br>In POPE<br>EMD-33396<br>PDB 7XQH	FIN, pH 8.0<br><br>In POPE<br>EMD-33397<br>PDB 7XQI	PLN, pH 8.0<br><br>In POPE<br>EMD-33398<br>PDB 7XQJ
**Data collection and processing**												
Magnification	96,000	96,000	92,000	120,000	96,000	96,000	105,000	105,000	105,000	105,000	105,000	105,000
Voltage (kV)	300	300	200	300	300	300	300	300	300	300	300	300
Electron exposure (e$^-$/Å^2)	40	40	40	40	40	40	72.42	72.42	72.42	72.42	72.42	72.42
Defocus range (μm)	–1.25 to –2.75	–1.25 to –2.75	–1.2 to –2.8	–1.25 to –2.75	–1.25 to –2.75	–1.25 to –2.75	–0.9 to –2.4	–0.9 to –2.4	–0.9 to –2.4	–0.9 to –2.4	–0.9 to –2.4	–0.9 to –2.4
Pixel size (Å)	0.675	0.675	1.144	0.5235	0.675	0.675	0.848	0.848	0.848	0.848	0.848	0.848
Number of micrographs	4,344	3,016	3,246	6,760	2,930	2,781	11,422	11,422	12,245	12,245	12,245	12,245
Symmetry imposed	D6	D6	D6	D6	D6	C6	D6	C1	C6	C6	C6	C6
Initial particle images (no.)	1,130,417	3,224,367	1,581,567	1,683,178	518,337	559,754	7,111,406	7,111,406	4,147,094	4,147,094	4,147,094	4,147,094
Final particle images (no.)	21,689	8,861	32,618	28,341	21,621	7,446	230,663	325,564	18,108	50,633	128,883	35,388
Map resolution (Å)	3.1	3.3	3.3	3.6	3.0	3.6	2.4	2.7	3.8	3.8	3.7	4.0
FSC threshold	0.143	0.143	0.143	0.143	0.143	0.143	0.143	0.143	0.143	0.143	0.143	0.143
Map resolution range (Å)	2.96-3.87	3.04-4.13	2.49-5.70	3.32-6.83	2.84-4.21	3.40-5.96	2.19-3.15	2.47-4.11	3.63-5.23	3.48-4.94	3.34-4.39	3.60-4.99
**Refinement**												
Initial model used (PDB code)	*De novo*	N/A	7F92	7F92	7F92	*De novo*	7F92	7F92	7F92	7F92	*De novo*	7F94
Model resolution (Å)	3.1	N/A	3.6	3.8	3.0	3.6	2.3	2.7	4.0	3.8	3.5	3.9
FSC threshold	0.5	N/A	0.5	0.5	0.5	0.5	0.5	0.5	0.5	0.5	0.5	0.5
Model resolution range (Å)	3.07-3.13	N/A	3.57-3.91	3.83-4.10	2.97-3.03	3.56-3.82	2.29-2.49	2.68-2.85	3.97-4.18	3.75-3.93	3.52-3.69	3.94-4.10
Map sharpening *B* factor (Å^2)	–38.129	–102.1	–139.2	–122.3	–99.6	N/A	Local	Local	N/A	N/A	N/A	N/A
Model composition												
Nonhydrogen atoms	21,240	N/A	19,632	20,808	22,812	19,128	23,172	23,012	10,206	9.774	9,582	9,960
Protein residues	2,352	N/A	2,412	2,352	2,424	2,352	2,412	2,412	1,206	1.200	1,176	1,224
Ligands	132	N/A	N/A	120	168	N/A	168	156	12	N/A	N/A	N/A
B factors (Å^2)												
Protein	30.17	N/A	125.21	119.79	42.07	132.54	59.74	86.27	174.86	159.97	138.23	174.61
Ligand	43.90	N/A	N/A	113.27	56.02	N/A	77.84	106.34	224.45	N/A	N/A	N/A
R.m.s. deviations												
Bond lengths (Å)	0.008	N/A	0.005	0.007	0.004	0.006	0.003	0.002	0.002	0.005	0.006	0.007
Bond angles (°)	0.954	N/A	0.845	0.942	0.534	0.939	0.517	0.472	0.458	0.762	0.740	0.854
**Validation**												
MolProbity score	1.16	N/A	1.61	1.73	1.10	1.47	1.14	1.10	1.78	1.83	1.59	2.36
Clashscore	0.64	N/A	1.72	3.42	2.77	3.62	3.54	3.01	4.64	5.33	5.57	6.15
Poor rotamers (%)	2.29	N/A	3.89	4.00	1.10	0.57	0.56	0.56	5.00	2.23	2.27	6.01
Ramachandran plot												
Favored (%)	96.96	N/A	96.45	97.22	97.98	95.49	98.35	97.97	97.97	95.92	97.92	93.50
Allowed (%)	3.04	N/A	3.55	2.78	2.02	4.51	1.65	2.03	1.52	3.57	2.08	6.50
Disallowed (%)	0	N/A	0	0	0	0	0	0	0.51	0.51	0	0

Supplementary Discussion

Potential role of cholesterols in the channel gating.

Compared to the Cx43-M257 GJIChs in the POPE nanodiscs (condition 8), those in LMNG/CHS contained more GCN, less PLN, and undetectable FIN protomers (condition 2), and those in POPE/CHS (condition 7) contained only GCN protomers. This suggests that a high CHS concentration (>10% of total lipids/detergents by mass) efficiently inhibits the PLN and FIN conformations and stabilizes the GCN conformation. Because the sterol ring of CHS is responsible for the interaction with the channel in the GCN conformation, cholesterol in the plasma membrane might play a crucial role in the gating of Cx43 GJICh; however, this has yet to be demonstrated experimentally. Studies using quantitative imaging analysis revealed that the available cholesterol concentrations in the inner and outer leaflets of the mammalian plasma membrane ($[Chl]_I$ and $[Chl]_O$) differed greatly[1, 2]. While $[Chl]_O$ concentration in HeLa cells was approximately 44% of the total lipids by mole (approximately 44 mol%), $[Chl]_I$ concentration was only approximately 3.6 mol%. In various cell types, $[Chl]_I$ concentration was generally low, ranging from 0.5 to 3.6 mol%. Because the membrane opening found in our study connects the inner leaflet and the pore inside, $[Chl]_I$ concentration, not $[Chl]_O$ concentration, would be effective. In addition, a very low CHS concentration in POPE nanodiscs (condition 8) may closely mimic the inner leaflet of the cell membrane in terms of the molar ratio of phospholipids and cholesterols. Therefore, Cx43 GJIChs in the cell membrane may have a conformational equilibrium similar to that in condition 8 (the 4:2:4 ratio of GCN, FIN, and PLN protomers), unless other cellular factors strongly affect the conformational equilibrium.

Considerable noise in four hemichannel sub-structures.

Four structures from dataset 8 (Supplementary Fig. 3c) were reconstructed from the hemichannel regions of the Cx43 GJICh particles. Therefore, one should be careful that they are not structures of undocked hemichannels in a single membrane. It should also be noted that all structures contain considerable noise map densities from alternative conformations. For example, in the distribution of PLN protomers in each hemichannel sub-particle (Fig. 4b),

approximately 16%, 6%, and 1% of hemichannel sub-particles had four, five, and six PLN protomers, respectively. While these sub-particles (approximately 23%) were expected to be classified into the PLN hemichannel sub-structure class, the 3D classification recognized only 14.3% of the sub-particles for the class, which probably contained 8%, 6%, and 1% sub-particles with four, five, and six PLN protomers, respectively. Therefore, the average structure made from the images of these hemichannel sub-particles would contain approximately 76% PLN and 24% other (GCN or FIN) protomers.

The FIN hemichannel sub-structure was reconstructed with 51.9% of the hemichannel sub-particle images, whereas the FIN protomer class included only 22% of the protomer images (Supplementary Fig. 3). Therefore, this structure contains substantial map densities from conformationally different protomers. While we identified high densities of FINs and PLNs, GCN densities were not detectable. However, the map densities of the TM helices were homogeneous and similar to those of the PLN hemichannel sub-structure (Supplementary Figs. 7a and 9c). If the structures of the TM helices in the FIN protomer were similar to those in the GCN and GCN^{TM1i} protomers, we should have been able to observe strong densities blocking membrane opening (Fig. 6a). Therefore, we are sure that the majority of FIN protomers have TM helices structurally similar to those of the PLN protomer and modeled TM helices based on the PLN protomer structure.

Evolutionary conservation of the structural changes in TM1 and NTH.

The dynamic π-helix in the middle of TM1 is one of three most conserved regions in human connexins (Supplementary Fig. 7b) and is almost identically conserved in classes A and B (Supplementary Fig. 7c). In addition, the cytoplasmic part of TM1 (residues 20–28), which rotates during the α-to-π-helix transition, is highly conserved in human connexins (Supplementary Fig. 7c). This suggests that other connexins may undergo the same structural changes as TM1 to not only regulate intercellular transport, but also create membrane openings. TM1 has another π-helix at the extracellular end, which is conserved in the Cx26 and Cx46/50 GJICh structures (Supplementary Fig. 7d). This π-helix remains unchanged between the GCN and PLN conformations (Fig. 5a). This sequence is highly conserved throughout the connexin family, indicating that the π-helix is structurally important for maintaining a 30° kink at the boundary between TM1 and ECL1.

The interaction of N-terminal tryptophan (W4) with the hydrophobic pocket formed by two TM1s and TM2 is crucial for PLN conformation (Supplementary Fig. 7e), and W4 is highly conserved in the connexin family. However, the hydrophobic residues involved in the intramolecular interaction between NTH and TM2, which are crucial for the conformation of GCN, are conserved only in class A and C connexins (Fig. 2d). Therefore, connexins in classes B and D may have different NTH conformations when dissociated from TMD.

Membrane opening.

A GJICh must discharge the lipids occluding its pores for channel opening during or after channel assembly. The only way for lipids to diffuse out of the channel would be between the two protomers, but it is unknown whether the two protomers are dissociated completely or only partly. Structural changes in the TM helices during the GCN-to-FIN transition resulted in the formation of a V-shaped membrane opening between two neighboring TMDs in Cx43 GJICh (Fig. 6a,b and Supplementary Fig. 10a), suggesting the partial dissociation in the cytoplasmic region of the TMD and potential lateral diffusion to the inner leaflet. This is consistent with the membrane opening in the innexin-6 hemichannel structure[3]. The surface of the membrane opening in Cx43 GJICh is formed mostly by hydrophobic and basic residues (Supplementary Fig. 10b) and is connected to the hydrophobic path to the CHS1-binding site in the FIN conformation. Some basic residues (K23, K109, and K234) and hydrophobic residues in this area are highly conserved in human connexins (Supplementary Fig. 10b).

To validate whether phospholipids can pass through the membrane openings in the PLN and FIN hemichannel sub-structures, we performed MD simulations based on the CHARMM36m force field[4] with corrections for charge-charge interactions[5, 6]. The channels were incorporated into bilayer systems of 1-palmitoyl-2-oleoyl-*sn*-glycero-3-phosphatidylcholine (POPC), and each system was simulated for 400 ns. We detected several phospholipid molecules partially penetrating in a headfirst fashion through the membrane opening in full PLN and FIN GJIChs (Supplementary Fig. 10d,e and Supplementary Movie 4) but not in full GCN GJICh (Supplementary Fig. 10c). Although we did not observe the complete penetration of a lipid, the simulation showed that the membrane opening was large enough for phospholipids to access the inside of the pore. The full penetration may require the conformational changes of NTHs which cannot be correctly predicted by MD simulation. In addition, slightly smaller or larger

openings were formed when the FIN protomer was positioned next to the GCN or PLN protomer (Fig. 6d), suggesting that a bigger opening may be formed during dynamic conformational changes of individual protomers.

Structural comparison between Cx43 and Cx46/50 GJIChs in the PLN conformation.

Cx46 and Cx50 preferentially adopt the PLN conformation in both detergent (n-decyl-β-D-maltoside) and lipid (dimyristoylphosphatidylcholine) environments. This may be because of the different environments, such as different detergents or lipids, and the absence of CHS. Alternatively, the NTH–TMD interaction in Cx46/50 GJICh may be stronger than that in Cx43 GJICh, and this channel may function mainly in the full PLN state. This idea is supported by our observation that the close hydrophobic interaction of F6 with F30 and I34 in Cx46/50 GJICh was absent in Cx43 GJICh because Cx43 has A6 instead of F6. Nevertheless, since the residues involved in the intramolecular interaction of NTH and TM2 in the Cx43 GCN conformation are highly conserved in Cx46 and Cx50, Cx46/50 likely undergoes the same conformational change as Cx43 during the initial channel opening.

The membrane opening in the structure of Cx46/50 GJICh is much narrower than that of Cx43 GJICh[7], suggesting that the PLN conformation may not be the conformation for putative lateral lipid diffusion. However, because a marked decrease in the protomer-protomer interactions is common between Cx43 and Cx46/50 GJIChs, Cx46/50 may have an intermediate state with a larger membrane opening, which is similar to the FIN conformation of Cx43.

Conformational changes in the CL during the GCN-to-PLN transition.

In the conformationally heterotypic channel reconstructed from dataset 2, we found a large map density surrounding the cytosolic protrusion of TM2 in the full PLN hemichannel region but not in the full GCN hemichannel region (Supplementary Fig. 8d). Although the density was not strong enough to determine the secondary structure or complete connectivity of the protein, it was clear that part of the map was connected to the N-terminus of TM3 (Supplementary Fig. 8e,f). This map region, which was the C-terminal region of CL (Supplementary Fig. 8g), closely interacted with Y17 of the NTH–TM1 loop and M100 of TM2, suggesting that this interaction

may contribute to the formation of the loop conformation in the PLN protomer. Although a large map density was also observed in the PLN protomer structures under conditions 3 and 8, it was not detected in all the GCN and FIN structures, indicating that the observed density was specific to the PLN conformation and was not caused by detergent or lipid environments.

While neutral pH and the partial deletion of CTD increase the PLN/GCN ratio, the underlying mechanism remains unclear. Since the PLN conformation is structurally related to the partial stabilization of CL, the two conditions may directly affect the structure of CL or the potential CL-CTD interaction to eventually stabilize the PLN conformation. However, the change in the conformational equilibrium may not be the major reason for gating regulation by pH because the pH gating is also greatly dependent on Ca^{2+}/calmodulin[8].

Structural differences between the GCN conformations of Cx43 and Cx31.3.

First, the superposition of Cx43 GJICh and the Cx31.3 hemichannel[9] led to the poor alignment of TM2 and NTH but not TM1 (Supplementary Fig. 5c). In particular, the cytoplasmic end of TM2 in Cx43 was displaced by approximately 6 Å away from the pore center, when compared to the position of that in Cx31.3 (Supplementary Fig. 5d). Second, the interaction networks between hydrophobic amino acid residues differed considerably. For example, in Cx31.3, F5 of the NTH interacted closely with the L96 of TM2, whereas the corresponding residues of Cx43 (A6 and M100) were distant from each other (Fig. 2c and Supplementary Fig. 5d). This conformational difference resulted in a large hole between two neighboring NTHs and TM2 at the cytoplasmic gate of Cx43 GJICh. Notably, in all the full GCN structures of Cx43 GJICh, this hole was filled with strong map densities that could be attributed to partially resolved detergent or lipid molecules (see below; Fig. 3e and Supplementary Fig. 5e). The map densities of CHS were also found in a deep hydrophobic pocket between NTH, TM1, and TM2 (see below; Fig. 3c). This was in contrast to the density feature presumed to be a phospholipid in the corresponding pocket of the Cx31.3 hemichannel.

Detailed structure of H-type interconnexon docking interactions.

Residues in ECL1 that participate in interconnexon docking interactions are well conserved in the connexin family, whereas those in ECL2 include two diversified residues that correspond

to H194 and T186 of Cx43 (Supplementary Fig. 12a,b). The two residues in ECL2 are grouped into three types (K/R-N, H, and the rest) and are called compatibility motifs because two hemichannels with the same motif type can dock together[10]. Several mutations in these residues are related to human diseases such as Charcot–Marie–Tooth and ODDD[11, 12]. In Cx46 GJICh belonging to the K/R-N type, R177 and the conserved aspartate D188 of one protomer interact with N185 of the opposite protomer (Supplementary Fig. 12b,d). However, in H-type connexins, including Cx43, the asparagine in the K/R-N type is replaced with histidine, and the lysine/arginine is changed to various amino acids (T186 for Cx43; Supplementary Fig. 12b,c). In the Cx43 GJICh structure, we found that H194 interacts with the conserved aspartate D197 in the opposite protomer, but not with T186 (Supplementary Fig. 12c). This interconnexon docking interaction in Cx43 GJICh is likely similar to that in Cx30.3, Cx31.9, Cx40, Cx45, and Cx47 GJIChs, which belong to the H-type[10]. In these H-type connexins, the residues corresponding to T186 in Cx43 are A163, A165, V184, V208 and P241, respectively. These residues would have been diversified because of no contribution in interconnexon docking interactions.

Mapping disease-causing mutations on the full GCN and PLN GJICh structures.

On the surface presentation of the full GCN and PLN GJICh structures, we mapped 14 missense mutations out of 25 selected mutations in Online Mendelian Inheritance in Man (OMIM) database (Supplementary Fig. 13). Although we could not elucidate their disease-causing mechanisms only with the structural information, we provide their potential structural effects on each conformation based on our observation of detailed interactions between amino acids (Supplementary Fig. 13). Remarkably, 9 of 14 missense mutations are mapped on the region undergoing large conformational changes during the GCN-to-PLN transition (Supplementary Fig. 13a,b; boxes 3 and 4), suggesting that defects in dynamic conformation changes may be the main cause for ODDD and keratodermia.

Supplementary References

1. Liu SL*, et al.* Orthogonal lipid sensors identify transbilayer asymmetry of plasma membrane cholesterol. *Nat Chem Biol* **13**, 268-274 (2017).
2. Buwaneka P, Ralko A, Liu SL, Cho W. Evaluation of the available cholesterol concentration in the inner leaflet of the plasma membrane of mammalian cells. *J Lipid Res* **62**, 100084 (2021).
3. Burendei B*, et al.* Cryo-EM structures of undocked innexin-6 hemichannels in phospholipids. *Sci Adv* **6**, eaax3157 (2020).
4. Huang J*, et al.* CHARMM36m: an improved force field for folded and intrinsically disordered proteins. *Nat Methods* **14**, 71-73 (2017).
5. Yoo J, Aksimentiev A. New tricks for old dogs: improving the accuracy of biomolecular force fields by pair-specific corrections to non-bonded interactions. *Phys Chem Chem Phys* **20**, 8432-8449 (2018).
6. Yoo J, Aksimentiev A. Improved Parameterization of Amine-Carboxylate and Amine-Phosphate Interactions for Molecular Dynamics Simulations Using the CHARMM and AMBER Force Fields. *J Chem Theory Comput* **12**, 430-443 (2016).
7. Flores JA*, et al.* Connexin-46/50 in a dynamic lipid environment resolved by CryoEM at 1.9 A. *Nat Commun* **11**, 4331 (2020).
8. Peracchia C. Chemical gating of gap junction channels; roles of calcium, pH and calmodulin. *Biochim Biophys Acta* **1662**, 61-80 (2004).
9. Lee HJ*, et al.* Cryo-EM structure of human Cx31.3/GJC3 connexin hemichannel. *Sci Adv* **6**, eaba4996 (2020).
10. Koval M, Molina SA, Burt JM. Mix and match: investigating heteromeric and heterotypic gap junction channels in model systems and native tissues. *FEBS Lett* **588**, 1193-1204 (2014).
11. Gong XQ, Nakagawa S, Tsukihara T, Bai D. A mechanism of gap junction docking revealed by functional rescue of a human-disease-linked connexin mutant. *J Cell Sci* **126**, 3113-3120 (2013).
12. Bai D, Wang AH. Extracellular domains play different roles in gap junction formation and docking compatibility. *Biochem J* **458**, 1-10 (2014).

Source Data of Supplementary Figure 1.

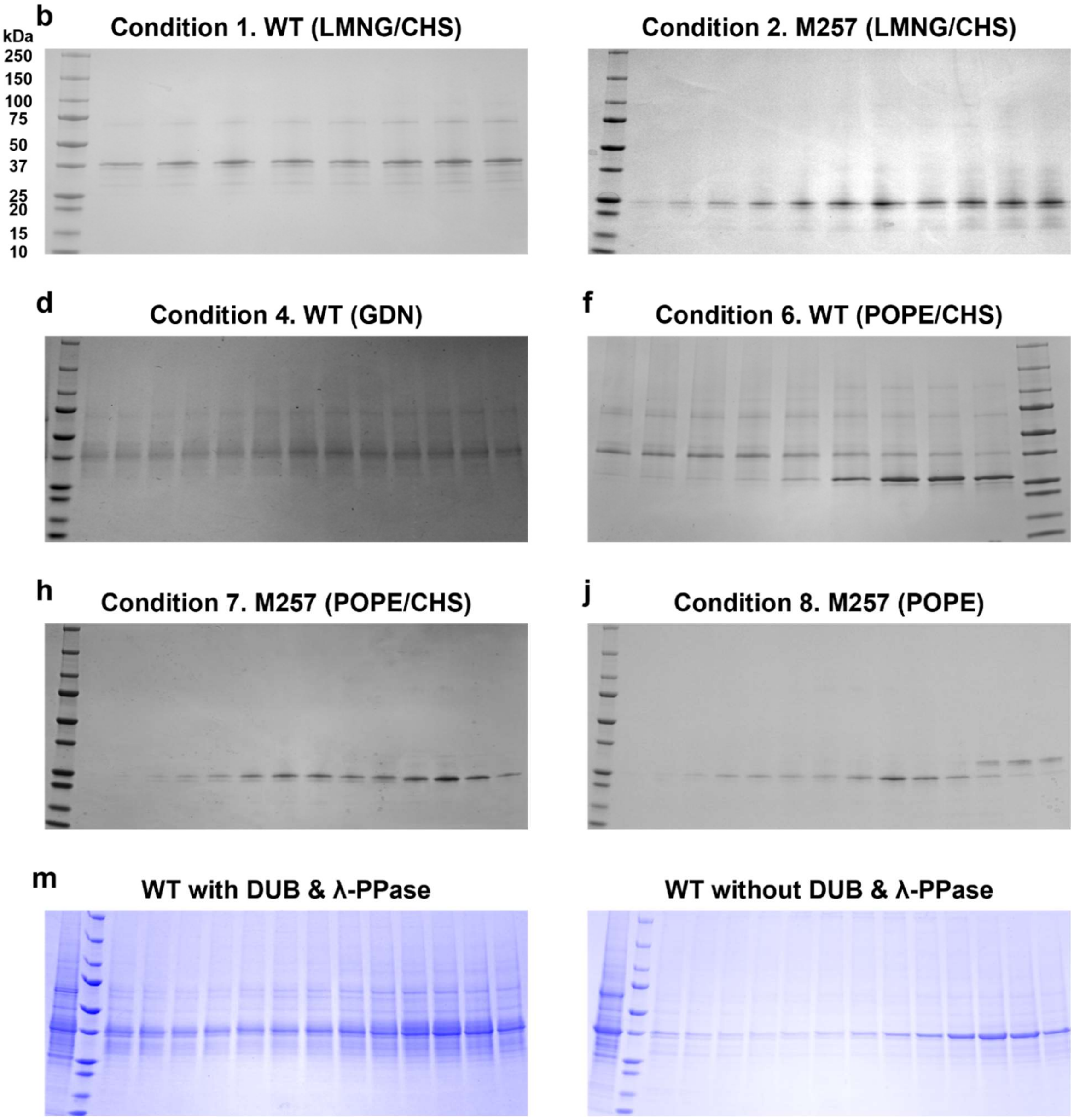

Source Data of Supplementary Figure 4.

To validate the Cryo-EM reconstruction maps for each dataset, we provided the angular distribution information. Abbreviations: con., condition; sym, symmetry.

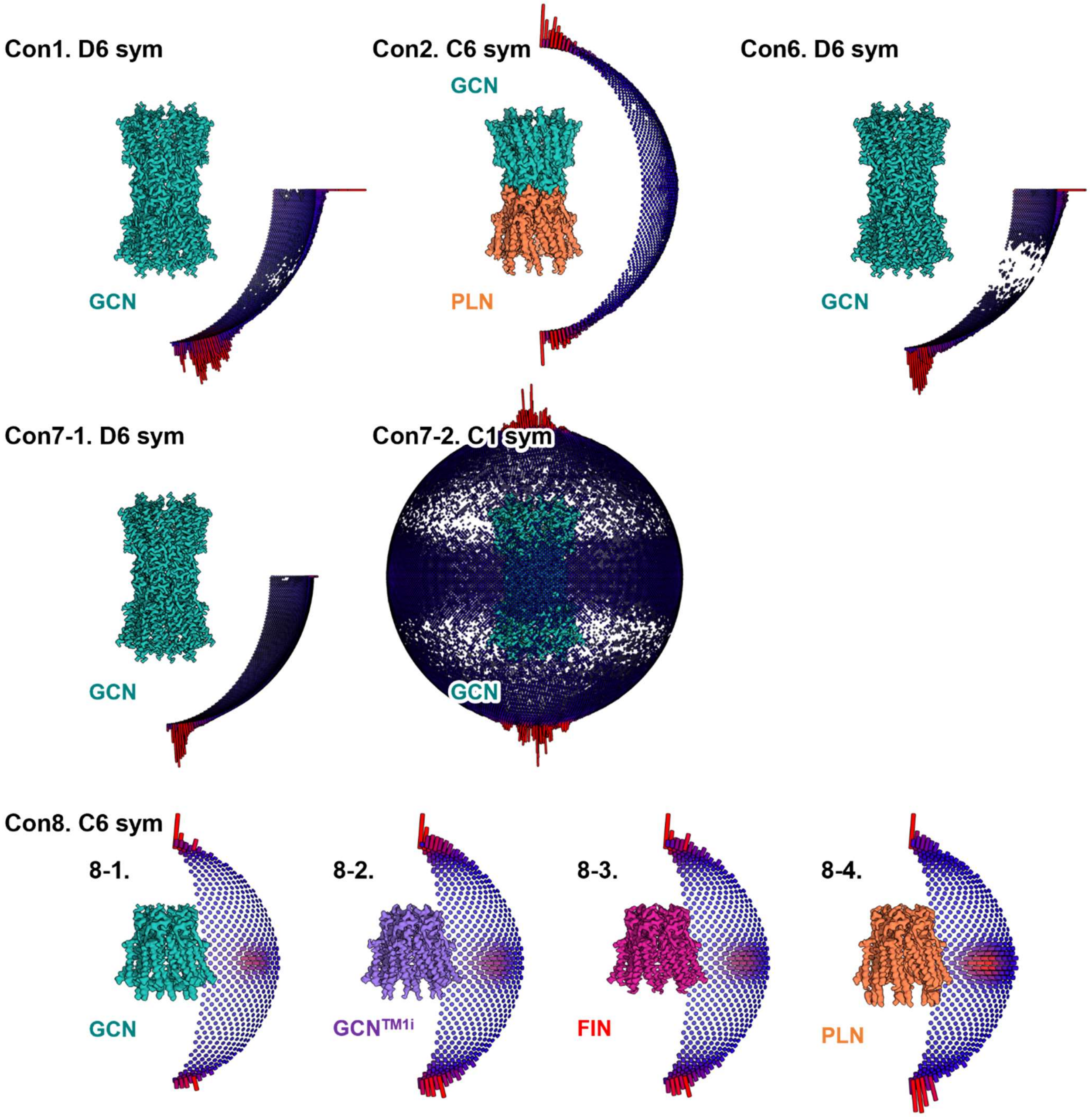

Supplement: Supplementary file 1 — Supplementary Information [file 41467_2023_36593_MOESM1_ESM.pdf]
