# Supplementary material for: Conformational changes in the human Cx43/GJA1 gap junction channel visualized using cryo-EM
Source: Nat Commun. 2023 Feb 18;14:931. doi: 10.1038/s41467-023-36593-y (PMC9938869; doi:10.1038/s41467-023-36593-y)
Supplement: Supplementary file 3 — Description of Additional Supplementary Files [file 41467_2023_36593_MOESM3_ESM.pdf]

File name: Supplementary Movie 1

Description: Structural change in Cx43.
Cx43 protomers in the GCN, FIN, and PLN conformations are colored in green, red, and orange, respectively. NTHs are colored in magenta. The conformational transition was visualized using the Morph tool in Chimera. Residues of NTH and TM1 undergoing substantial structural changes are drawn as sticks and labeled.

File name: Supplementary Movie 2

Description: Creation of the membrane opening during the GCN-to-PLN transition.
Structural changes in the intermolecular interface during the transition between GCN and PLN conformations were visualized using the Morph tool in Chimera. Residues involved in the close intermolecular interaction in GCN hemichannel are drawn as sticks and labeled. NTHs are colored in magenta.

File name: Supplementary Movie 3

Description: Changes in the interaction between the NTH-TM1 loop and TM helices during the GCN-to-PLN transition.
Structural changes were visualized using the Morph tool in Chimera. NTH and the NTH-TM1 loop are colored in magenta. Residues participating in the interaction between the NTH-TM1 loop and TM helices are drawn as sticks and labeled.

File name: Supplementary Movie 4

Description: Partial penetration of a phospholipid through the membrane opening between FIN protomers.
Partial penetration of a POPC molecule through the membrane opening was observed during the MD simulation of the FIN hemichannel sub-structure in a phospholipid bilayer system. The FIN hemichannel sub-structure is drawn as cartoon. The selected POPC is drawn as sticks and colored in cyan. Other lipids in the lipid bilayer are omitted for clarity.
