# Supplementary material for: Conformational changes in the human Cx43/GJA1 gap junction channel visualized using cryo-EM
Source: Nat Commun. 2023 Feb 18;14:931. doi: 10.1038/s41467-023-36593-y (PMC9938869; doi:10.1038/s41467-023-36593-y)
Supplement: Supplementary file 8 — Reporting Summary [file 41467_2023_36593_MOESM8_ESM.pdf]

Corresponding author(s): Jae-Sung Woo
Last updated by author(s): Jan 25, 2023

Reporting Summary

Nature Portfolio wishes to improve the reproducibility of the work that we publish. This form provides structure for consistency and transparency in reporting. For further information on Nature Portfolio policies, see our Editorial Policies and the Editorial Policy Checklist.

Statistics

For all statistical analyses, confirm that the following items are present in the figure legend, table legend, main text, or Methods section.

n/a	Confirmed	
☐	☒	The exact sample size (*n*) for each experimental group/condition, given as a discrete number and unit of measurement
☐	☒	A statement on whether measurements were taken from distinct samples or whether the same sample was measured repeatedly
☐	☒	The statistical test(s) used AND whether they are one- or two-sided<br>*Only common tests should be described solely by name; describe more complex techniques in the Methods section.*
☒	☐	A description of all covariates tested
☒	☐	A description of any assumptions or corrections, such as tests of normality and adjustment for multiple comparisons
☐	☒	A full description of the statistical parameters including central tendency (e.g. means) or other basic estimates (e.g. regression coefficient) AND variation (e.g. standard deviation) or associated estimates of uncertainty (e.g. confidence intervals)
☒	☐	For null hypothesis testing, the test statistic (e.g. *F*, *t*, *r*) with confidence intervals, effect sizes, degrees of freedom and *P* value noted<br>*Give P values as exact values whenever suitable.*
☒	☐	For Bayesian analysis, information on the choice of priors and Markov chain Monte Carlo settings
☒	☐	For hierarchical and complex designs, identification of the appropriate level for tests and full reporting of outcomes
☒	☐	Estimates of effect sizes (e.g. Cohen's *d*, Pearson's *r*), indicating how they were calculated

Our web collection on statistics for biologists contains articles on many of the points above.

Software and code

Policy information about availability of computer code

Data collection	EPU v.1.11.1.50
Data analysis	RELION 3.1, cryoSPARC 3.1, MotionCor 2 v.1.2.6., Gctf v.1.18, ResMap v.1.1.4, Coot 0.9.6, PHENIX 1.19.2, MolProbity v.4.5.2, HOLE v.2.2.005, CAVER v.3.03, GROMACS 2020.2, CHARMM36m, UCSF Chimera v.1.13

For manuscripts utilizing custom algorithms or software that are central to the research but not yet described in published literature, software must be made available to editors and reviewers. We strongly encourage code deposition in a community repository (e.g. GitHub). See the Nature Portfolio guidelines for submitting code & software for further information.

Data

Policy information about availability of data

All manuscripts must include a data availability statement. This statement should provide the following information, where applicable:

- Accession codes, unique identifiers, or web links for publicly available datasets
- A description of any restrictions on data availability
- For clinical datasets or third party data, please ensure that the statement adheres to our policy

Atomic coordinates and cryo-EM density maps have been deposited in PDB and EMDB as follows: Cx43-WT GJICh (pH 8.0, LMNG/CHS) in full GCN conformation, PDB 7F92 and EMD-31495; Cx43-M257 GJICh (pH 8.0, LMNG/CHS) with two conformationally different hemichannels, PDB 7F94 and EMD-31497; Cx43-WT GJICh (pH 8.0, GDN) in full GCN conformation, PDB 7XQ9 and EMD-33391; Cx43-WT GJICh (pH 8.0, Soybean lipids) in full GCN conformation, PDB 7F93 and EMD-31496;

Cx43-WT GJICh (pH 8.0, POPE/CHS) in full GCN conformation, PDB 7XQB and EMD-33392; Cx43-M257 GJICh (pH 8.0, POPE/CHS) in full GCN conformation, PDB 7XQF and EMD-33394; Cx43-M257 GJICh (pH 8.0, POPE/CHS, C1 symmetry) in full GCN conformation, PDB 7XQD and EMD-33393; hemichannel-focused structure of Cx43-M257 GJICh (pH 8.0, POPE) in full GCN conformation, PDB 7XQG and EMD-33395; hemichannel-focused structure of Cx43-M257 GJICh (pH 8.0, POPE) in full GCNTM1i conformation, PDB 7XQH and EMD-33396; hemichannel-focused structure of Cx43-M257 GJICh (pH 8.0, POPE) in full FIN conformation, PDB 7XQI and EMD-33397; hemichannel-focused structure of Cx43-M257 GJICh (pH 8.0, POPE) in full PLN conformation, PDB 7XQJ and EMD-33398. The consensus cryo-EM density map of Cx43-WT GJICh (pH 6.9, LMNG/CHS) has been deposited in EMDB with ID EMD-33399. MD trajectories have been deposited to Zenodo (https://doi.org/10.5281/zenodo.7219679).

Human research participants

Policy information about studies involving human research participants and Sex and Gender in Research.

Reporting on sex and gender	n/a
Population characteristics	n/a
Recruitment	n/a
Ethics oversight	n/a

Note that full information on the approval of the study protocol must also be provided in the manuscript.

Field-specific reporting

Please select the one below that is the best fit for your research. If you are not sure, read the appropriate sections before making your selection.

☒ Life sciences ☐ Behavioural & social sciences ☐ Ecological, evolutionary & environmental sciences

For a reference copy of the document with all sections, see nature.com/documents/nr-reporting-summary-flat.pdf

Life sciences study design

All studies must disclose on these points even when the disclosure is negative.

Sample size	For cryo-EM, sufficient sample size varies from protein to protein and cannot be pre-calculated before the data collection. We collected the data until we obtained the quality of 3D reconstruction maps that reaches the overall resolution better than 4.0 angstrom. Poor quality or insufficient number of particle images cannot produce a reliable 3D reconstruction maps, which can be assessed by the connectivity of the main chain map densities and the identification of individual amino acids based on side-chain map densities. Since all presented structures in this study were determined at very high resolutions over 4 angstrom, they have sufficiently good main and side-chain map densities to build reliable atomic models. We also provided Fourier Shell Correlation (FSC) curves between the two unfiltered half maps and between the model and the full map, which are standard quality measures for cryo-EM maps.
Data exclusions	Particle images lacking high resolution features in 2D and 3D classifications were excluded. Those images contain damaged proteins, proteins in thick ice, impurities or no proteins, and thus need to be excluded to obtain a higher resolution structure. Since individual particle images have very low resolution, investigators cannot select out particles with a specific protein conformation, and thus this process cannot be intentional.
Replication	All sample preparation procedures for cryo-EM experiments were carried out at least in triplicate and attempts were successful. Experiments for purification were also replicated more than three times with similar results.
Randomization	In each cryo-EM experiment, particle images were randomly splitted into two groups by a computer program for calculating Fourier-shell correlation coefficients. For the final 3D reconstruction, we did not allocate particle images into groups but used all to obtain higher resolution and better quality of the map density.
Blinding	Blinding was not utilized. No experiment on human or animals was included in this study. The reliability of each structure was evaluated by density map quality and resolution. The full process of the cryo-EM study could not be repeated by others, since it would require the dedication of highly qualified researchers for 6 to 12 months for each structure.

Reporting for specific materials, systems and methods

We require information from authors about some types of materials, experimental systems and methods used in many studies. Here, indicate whether each material, system or method listed is relevant to your study. If you are not sure if a list item applies to your research, read the appropriate section before selecting a response.

Materials & experimental systems

n/a	Involved in the study
☐	☒ Antibodies
☐	☒ Eukaryotic cell lines
☒	☐ Palaeontology and archaeology
☒	☐ Animals and other organisms
☒	☐ Clinical data
☒	☐ Dual use research of concern

Methods

n/a	Involved in the study
☒	☐ ChIP-seq
☒	☐ Flow cytometry
☒	☐ MRI-based neuroimaging

Antibodies

Antibodies used	rho-1D4 antibody was purchased from University of British Columbia and conjugated with agarose resin to purificate proteins with 1D4 epitopes.
Validation	rho-1D4 antibody have been validated by the manufacturer and numerous experiments with different proteins performed by researchers worldwide.

Eukaryotic cell lines

Policy information about cell lines and Sex and Gender in Research

Cell line source(s)	Human embryonic kidney (HEK) 293E cells are obtained from ATCC.
Authentication	Cell lines were not authenticated.
Mycoplasma contamination	HEK293E cells were tested negative for mycoplasma contamination.
Commonly misidentified lines (See ICLAC register)	No commonly misidentified cell line was used in this study.
